# Sustainable Athletes’ Career Pathways and Mental Health Support: An Integrative Umbrella Review

**DOI:** 10.3390/sports14060251

**Published:** 2026-06-19

**Authors:** Francesca Di Rocco, Cristian Romagnoli, Simone Ciaccioni, Sabrina Demarie, Mojca Doupona, Laura Capranica, Elvira Padua, Flavia Guidotti

**Affiliations:** 1 Department of Human Sciences and Promotion of Quality of Life, “San Raffaele” Open University of Rome, 00166 Rome, Italy; francesca.dirocco@uniroma5.it (F.D.R.); cristian.romagnoli@uniroma5.it (C.R.); elvira.padua@uniroma5.it (E.P.); 2 Department of Movement, Human and Health Sciences, University of Rome “Foro Italico”, 00135 Rome, Italy; simoneciaccioni@yahoo.it (S.C.); laura.capranica@uniroma4.it (L.C.); 3 Department of Sport Sociology, Faculty of Sport, University of Ljubljana, 1000 Ljubljana, Slovenia; mojca.doupona@fsp.uni-lj.si

**Keywords:** career transitions in elite sport, athletic identity, transition planning, mental health support, retirement adaptation

## Abstract

The present integrative umbrella review aims to provide a comprehensive overview of the evidence and practices related to mental health and career transitions in elite sport toward the implementation of service provision through digital interventions. Following PRIO guidelines, an extensive search across five databases (2015–2025) identified 52 eligible manuscripts (e.g., conceptual, review, and position studies). Data extraction focused on mental health, dual-career pathways, career transition challenges and needs, and identity-related issues among high-performance athletes. The findings revealed a strong consensus that athlete well-being is shaped by the dynamic interaction of mental health symptoms, sport-specific stressors, identity processes, and structural conditions across the athletic lifespan. Mental health vulnerabilities (e.g., anxiety, depression, disordered eating, and distress) were consistently reported, particularly during injury, deselection, and retirement. Dual-career engagement, diversified identities, and proactive career planning emerged as key protective factors, while stigma, limited literacy, and uneven access to psychological services remained persistent barriers. Five main thematic areas (Matrix 1) operationalized in ten higher-order intervention domains (e.g., Matrix 2, screening, monitoring, literacy, and others) and 14 potential online implementation strategies (Matrix 3) were identified. However, the evidence highlights fragmented implementation and a lack of scalable, cross-national tools to support athletes during and beyond their competitive careers. Therefore, a harmonized, evidence-based, multidimensional framework for the development and implementation of digital support resources has been proposed. This integrative review underscores the need for integrated, culturally sensitive, and digitally enabled support systems to promote sustainable transitions and long-term athlete well-being.

## 1. Introduction

Elite sport represents a high-performance ecosystem characterized by intense physical, psychological, and social demands [1]. While the pursuit of excellence is central to elite sport culture, the long-term well-being of athletes has become an increasingly prominent concern in both research and policy, particularly during the transition out of competitive sport [2,3]. In fact, retirement from elite sport is now widely recognized as a complex, multifactorial, and often destabilizing process involving profound changes in identity, mental health, social belonging, and vocational direction [4,5]. These transitions unfold within broader sustainability debates in sport, where long-term athlete welfare, equitable access to support, and systemic responsibility are central pillars [6,7,8]. As such, understanding and supporting sustainable career transitions is not only a matter of individual adaptation but also a structural imperative for sport organizations, educational institutions, policy bodies, and labor market stakeholders [1,3,9,10,11,12].

Over the past decade, a substantial body of research has examined the determinants of successful career transitions, the vulnerabilities associated with elite sport participation, and the organizational responsibilities of sport systems, consistently highlighting the central role of athletic identity and the risks associated with identity foreclosure, particularly when athletes define themselves primarily through their sporting role [13,14]. Identity foreclosure is strongly associated with maladaptive adjustment, emotional distress, and difficulties envisioning a meaningful life beyond sport. Conversely, diversified identities, dual-career engagement, and proactive career planning are repeatedly identified as protective factors that facilitate smoother transitions and enhance long-term well-being [12,15]. Furthermore, mental health has emerged as a particularly salient concern within this literature. Review-level evidence indicates that elite athletes experience symptoms of anxiety, depression, disordered eating, and psychological distress at rates comparable to or exceeding those of the general population [16,17]. These vulnerabilities are often exacerbated during career transitions, especially when retirement is abrupt, involuntary, or poorly supported [18,19]. In response, international bodies such as the International Olympic Committee have issued consensus statements emphasizing prevention, early identification, and culturally sensitive mental health services embedded throughout the athletic career [16,20,21]. Despite these advances, review-level analyses still reveal persistent gaps in implementation, uneven access to services, and enduring stigma surrounding help-seeking [22,23].

Parallel streams of research underscore the importance of dual-career pathways and educational engagement as mechanisms for promoting sustainable transitions. Athletes who pursue academic or vocational development alongside their sporting careers demonstrate greater psychological resilience, stronger social networks, and improved employability during and after sport [14,24,25]. However, structural inequities in the availability and quality of dual-career support have been highlighted, with significant variation across countries, sports, and demographic groups [26,27]. These disparities underscore the need for harmonized, cross-national frameworks capable of supporting diverse athlete populations.

Despite the richness of the existing literature, the evidence base remains fragmented. Many reviews focus on specific subdomains (e.g., mental health, dual careers, cultural perspectives, or retirement adjustment) without integrating findings across these interconnected areas. As a result, the field still lacks a consolidated, higher-order synthesis capable of identifying convergent themes, methodological consistencies, and persistent gaps across the existing literature. This fragmentation limits the ability of sport systems to design coherent, evidence-based support structures that align conceptual frameworks with practical interventions. This gap became particularly evident in our systematic literature review (SLR) of 117 primary studies on sustainable career transitions and mental health support in elite sport [3], which revealed substantial heterogeneity in research designs, populations, and conceptual models, alongside recurring challenges such as identity loss, limited transition planning, inconsistent access to psychological services, and uneven implementation of dual-career policies. Importantly, the study also identified 67 review-level, conceptual, and position manuscripts that fell outside the scope of the primary study synthesis but were deemed highly relevant for a broader, integrative understanding of the field. These manuscripts represent a rich yet under-synthesized body of knowledge capable of illuminating overarching patterns, conceptual convergences, and systemic gaps. In this framework, an integrative umbrella review could provide a valuable complementary contribution to further substantiate the sustainable development of digital support services to meet athletes’ needs in relation to mental health and career transitions. This hybrid approach allows for the structured synthesis of existing tertiary reviews (systematic and scoping reviews) while simultaneously incorporating vital contextual, theoretical, and normative evidence derived from narrative reviews, conceptual manuscripts, and position statements. In particular, umbrella reviews (e.g., systematic reviews of review manuscripts) offer a methodological approach that is particularly well suited to this task. By synthesizing review-level evidence, umbrella reviews provide a panoramic perspective on complex topics, identify areas of consensus and divergence, and highlight structural gaps that may not be visible within individual reviews. However, traditional umbrella reviews strictly synthesize systematic reviews and meta-analyses, potentially omitting critical context, foundational theories, and institutional directives. Accordingly, an integrative approach may provide a more holistic synthesis that aligns empirical intervention outcomes with current theoretical paradigms and expert consensus in the field. In this context, an integrative umbrella review can complement our SLR [3] and further clarify how existing evidence aligns with conceptual frameworks and practical interventions, and where misalignments persist. This is essential for guiding the development of sustainable, integrated, and digitally enabled support systems that respond to the evolving needs of elite athletes.

The sport sector is undergoing a rapid digital transformation [28,29]. Digital interventions such as e-counseling platforms, mobile applications, and online transition portals are increasingly recognized as scalable, cost-effective tools for delivering psychological support, career guidance, and educational resources to elite athletes [30]. Within this context, the ERASMUS+ “Supporting Olympians Transitioning to Real Life in Concern of their Mental Health” (PORTAL) project [31] exemplifies this shift through the development of a digital platform and a network of Real-Life Transition Officers to support Olympians facing retirement-related challenges. Such initiatives highlight the urgent need to align evidence, conceptual frameworks, and practical interventions within elite sport support systems. Without a consolidated understanding of the review-level evidence, digital and hybrid support models risk being developed in isolation from the broader scientific and policy landscape.

Therefore, the present integrative umbrella review aims to synthesize the review-level, conceptual, and position statement literature on (a) career transitions and retirement processes, (b) mental health challenges and protective factors, (c) dual-career and educational pathways as effective strategies for fostering successful retirement from sport, and (d) organizational, cultural, and systemic determinants of elite athletes’ well-being. By integrating findings across the review manuscripts identified in our SLR [3], this study seeks to provide a comprehensive, transdisciplinary understanding of sustainable career transitions in elite sport. Together, our SLR [3] and the present integrative umbrella review form a two-phase research program designed to inform the development of long-term, evidence-based, and digitally supported strategies for athlete mental health and career assistance, thereby contributing to the broader transformation of elite sport support systems. Accordingly, the present study also proposes a harmonized, evidence-based framework for comprehensive digital support addressing elite athletes’ mental health and career transition challenges and needs.

## 2. Materials and Methods

### 2.1. General Study Design Information and Protocol Registration

The European ERASMUS+ Sport Lump Sum Grant “SupPorting OlympiAns Transitioning to Real Life in concern in their mental health—PORTAL” project (101184857-ERASMUS-SPORT-2024-SCP) supported the present study. In considering that the study was conceived within a European-funded project, all participating project partners from different countries and representing different academic (e.g., San Raffaele Open University of Rome), education and innovation (e.g., Sport Innovation Hub, Spain; Stichting European Network For Innovation And Knowledge, the Netherlands), and sports organizations (e.g., National Olympic Committees: Croatia, Romania, North Macedonia; major national sport body: INSEP, France) contributed to the development and implementation of a predefined methodology (e.g., formalized in the project’s first deliverable: Methodological toolkit). Further, to enhance the methodological rigor and transparency, the review protocol was registered in the International Prospective Register of Systematic Reviews (PROSPERO, ID: CRD420251145490, available online: https://www.crd.york.ac.uk/PROSPERO/view/CRD420251145490—accessed on 20 April 2026).

Regarding the protocol adherence, the initial protocol was amended to produce two main outcome papers:

1. An SLR of primary research articles [3];
2. An integrative umbrella review, including review-level manuscripts.

This approach was considered appropriate to ensure that the 67 manuscripts excluded from our SLR narrative (e.g., including narrative reviews, scoping and systematic reviews, conceptual manuscripts, and position stand papers) could be addressed through a dedicated manuscript (the present study) as a relevant source of information for the development of sustainable digital support tools for elite athletes.

No substantive deviations occurred from the registered PROSPERO protocol. Two minor procedural refinements emerged during the review process: (a) the inclusion of conceptual manuscripts and position statements was more extensive than initially foreseen, reflecting their prevalence and relevance in this field; and (b) the thematic synthesis evolved into the development of structured outcome matrices to systematically integrate heterogeneous evidence types. This approach was deemed relevant to highlight key aspects that should be addressed when developing and implementing sustainable, evidence-based support services, thereby translating scientific evidence into real-life interventions. These refinements did not alter the core objectives, eligibility criteria, or analytic logic of the protocol but enhanced the methodological coherence of the integrative umbrella review. A detailed comparison between the registered PROSPERO protocol and the final review design is provided in Supplementary Materials File S1. This table documents the two minor procedural refinements that emerged during the review process.

### 2.2. Eligibility Criteria

Given the multidimensional and practice-oriented nature of elite athletes’ mental health and career transition support, the present integrative umbrella review adopted a hybrid, integrated evidence synthesis approach, allowing the inclusion of different forms of review-level, conceptual, practice-oriented, and expert literature. This integrative methodology is considered appropriate when the aim is to consolidate heterogeneous forms of knowledge into a coherent higher-order understanding of complex phenomena [32,33]. Importantly, the different study types included in this review were not treated as equivalent sources of evidence. Review-level papers contributed to aggregated empirical findings, whereas consensus and position statements provided expert-derived recommendations and organizational guidance. Finally, conceptual manuscripts offered theoretical and interpretive frameworks. Specific considerations are provided in Section 2.6.

The present integrative umbrella review was based on a modified version of the Preferred Reporting Items for Overviews of systematic reviews (PRIO) guidelines [34] (Supplementary Materials File S1), a dedicated guideline developed to enhance transparency, completeness, and methodological rigor in overviews of systematic reviews. PRIO builds on the conceptual structure of the Preferred Reporting Items for Systematic Reviews and Meta-analyses—PRISMA guidelines (PRISMA), sharing core principles such as explicit eligibility criteria, reproducible search strategies, structured data extraction, and transparent reporting of study selection. However, PRIO extends beyond PRISMA by addressing methodological challenges unique to umbrella reviews, including the appraisal of review-level evidence rather than primary studies, the management of overlapping primary studies across included reviews, and the interpretation of discordant findings between reviews. PRIO also requires detailed reporting of review characteristics, synthesis methods tailored to higher-order evidence integration, and explicit consideration of how methodological quality and overlap influence the conclusions of the overview. By combining the foundational transparency of PRISMA with the specialized requirements of PRIO, this methodological approach ensures robust reporting standards appropriate for synthesizing complex, multi-review evidence in the context of mental health and career support in elite sport.

The inclusion criteria for the studies encompassed: (i) a peer-reviewed systematic literature review (SLR), scoping review (ScR), and narrative review (NR) of manuscripts; (ii) conceptual manuscripts that did not meet the inclusion criteria of our previous SLR paper (Di Rocco et al.) [3]; (iii) consensus statements and position stand manuscripts pertaining to mental health and career assistance support in elite sport; (iv) scientific contributions published from January 2015 up to March 2025 in English; and (v) content relevance to the research objectives (e.g., addressing mental health, career, and retirement challenges and needs of elite athletic populations previously described in Di Rocco et al.) [3]. The exclusion criteria were: (i) primary research articles (identified and retained in our SLR manuscript [3]); (ii) not peer-reviewed publications; and (iii) out of scope and/or not relevant (e.g., involving youth, sub-elite, or student athlete populations; psychiatric disorders).

In the present study, the broader category of the student athlete population was excluded, in line with our previous SLR [3] and with the objectives of the PORTAL project, focusing specifically on elite, high-performance, and Olympic-level athletes. Although dual-career research frequently involves student athletes, their developmental stage, institutional structures, and academic–sport balance differ substantially from those of elite athletes navigating professional or post-elite transitions. For this reason, only dual-career evidence pertaining to elite or professional athletes was retained, ensuring conceptual coherence with the target population of the review.

### 2.3. Literature Search Process

A systematic literature search was conducted across five databases: SPORTDiscus (EBSCOhost), PsycINFO, Scopus, Web of Science, and Google Scholar. Original articles were retained for our SLR [3], whereas conceptual and review manuscripts were considered for the present integrative umbrella review.

The search strategy was developed iteratively and refined through pilot testing across databases to ensure correct Boolean operator precedence and optimal sensitivity. The final search syntax employed a fully nested Boolean structure, for example:

“((“elite athlete” OR “high-performance athlete” OR Olympic athlete) AND (“career transition” OR retirement OR “dual career”) AND (“mental health” OR “psychological distress” OR wellbeing OR “psychological support”))*”.

Database-specific controlled vocabulary was incorporated where available (e.g., APA Thesaurus terms in PsycINFO and MeSH terms in PubMed-indexed records; SPORTDiscus Subject Headings; Scopus-indexed keywords). Truncation (*) and wildcard symbols were adapted to each database’s requirements. The complete database-specific search strings are reported in Supplementary Materials File S1. The search was conducted between January and March 2025, with automated alerts activated until May 2025 to capture newly published material. Backward and forward citation tracking was conducted for all included manuscripts to ensure comprehensive coverage.

To note, because conceptual manuscripts, consensus statements, and position papers are inconsistently indexed across databases and often lack standardized terminology, these document types could not be reliably retrieved through controlled vocabulary or Boolean keyword combinations. Pilot testing confirmed that terms such as “consensus statement”, “position stand”, “framework”, or “conceptual model” did not consistently identify relevant documents and frequently generated large numbers of irrelevant records. For this reason, and in accordance with PRISMA and PRIO recommendations for identifying non-indexed or poorly indexed literature, these document types were primarily identified through backward and forward citation tracking and manual screening during full-text assessment [35]. Their inclusion was therefore ensured through complementary search techniques rather than keyword-based retrieval, which would have reduced sensitivity and compromised reproducibility.

Verification of duplicate entries was conducted in Excel (version 2017, Microsoft Corp., Redmond, WA, USA) to manage overlapping records. In particular, key bibliographic fields (i.e., title, first author, year, and DOI) and manual validation were used.

### 2.4. Study Selection and Data Collection Process

The screening process was conducted independently by three authors (F.D.R., F.G., and C.R.) with strong expertise in sport sciences and dual-career pathways, and who were members of the PORTAL project team. These reviewers examined each record based on title, keywords, abstract, and full text, documenting exclusion decisions and ensuring that all retained articles met the predefined eligibility criteria and aligned with the objectives of the study. The pool of eligible articles was evenly allocated among four reviewers, with each article undergoing independent assessment by two of them. Any discrepancies between the two reviewers were resolved through consultation with a fifth author (S.C.). When disagreements persisted after this step, a sixth senior author (L.C.) was consulted to reach a final decision on eligibility.

### 2.5. Manuscripts’ Appraisal and Confidence of Evidence Evaluation

In considering the different study typologies eligible for the present study, several tools were applied to conduct the appraisal of the included manuscripts (Supplementary Materials File S2).

To ensure a rigorous and transparent evaluation of included manuscripts, SLRs and ScRs were assessed through the AMSTAR 2 [36] and JBI tools [37], respectively. Both tools are highly recommended for umbrella reviews. In particular, AMSTAR 2 was developed to assist scholars in the assessment and identification of systematic reviews meeting high quality standards, including those based on non-randomized studies. The tool includes 16 items to be rated with positive (e.g., Yes; Partial yes), negative (e.g., No), or Not applicable (e.g., NA) answers, leading to an overall confidence judgment. Notably, overall rating depends on whether weaknesses occur in critical and/or non-critical domains (e.g., Critically low: more than one critical flaw OR one critical flaw and multiple non-critical weaknesses; Low: one critical flaw but only a small number of non-critical weaknesses; Moderate: no critical flaws but more than one non-critical weakness; High: no critical flaws and no more than one non-critical weakness). Similarly, the JBI assessment tool includes 11 items to be rated with positive (e.g., Yes; Partial yes), negative (e.g., No), Unclear, and Not applicable (e.g., NA) answers to evaluate the extent to which a study has addressed the possibility of bias in its design, conduct, and analysis, leading to an overall quality judgment (e.g., Low, Moderate, High). Both AMSTAR 2 and JBI tools align with the PRISMA criteria, encompass rigorous appraisal criteria specific to the study typology (e.g., systematic reviews and meta-analyses, and scoping reviews, respectively), appraise both methodological quality and risk of bias to ensure evidence reliability to guide decision-makers, and support evidence-based practice. Although both tools are consistently applied in clinical research, especially when the assessment of the efficacy of clinical interventions is required, their application in a broader research area (e.g., such as the present integrative umbrella review, pertaining to the multidimensional assessment of possible interventions to be implemented to assist elite athletes’ mental health and career management and transitions) may present limitations [38]. In fact, many systematic reviews in sport psychology and social science do not include meta-analyses, randomized trials, or preregistered protocols, which are central AMSTAR 2 criteria. Therefore, the integration of the appraisal of included SLRs and ScRs was considered crucial to ensure both quality and scientific rigor were met before data extraction and to avoid over-penalizing studies for criteria that are structurally irrelevant in social science research. Hence, an adapted version of the Quality Assessment Tool for Reviews (QATR) [39] was also used to assess the methodological quality of the included SLRs and ScRs. The instrument was considered suitable and reliable for appraisal evaluation in several research contexts [40] and mainly focuses on general methodological quality standards for the evaluation of a wide range of studies. Eight quality criteria questions were applied, with 1 point (pt.) assigned to positive (e.g., “Yes”) answers, and 0 pt. to negative (e.g., “No”), or doubtful (e.g., “Can’t tell”) ones, respectively. Then, positive answers assigned one point to the evaluated manuscript, which could yield a quality score ranging from 0 to 8 pt (e.g., labels: weak, range: 0–3 pt; moderate, range: 4–6 pt; or strong, range: 7–8 pt).

The Scale for the Assessment of Narrative Review Articles (SANRA) [41] was applied to evaluate NR manuscripts. In particular, the tool was developed as a brief critical appraisal instrument for evaluating the construct quality of non-systematic review studies, including six items, to be rated from 0 pt to 2 pt, leading to a maximal summative score of 12 pt regarding studies’ construct quality (e.g., 1–4 pt = weak; 5–8 pt = moderate; 9–12 pt = strong) [40]. The same tool was also applied to assess conceptual manuscripts, excluding item 3 (e.g., Description of the literature search: Does the article mention the databases searched, search terms, or the types of literature included?), leading to a maximum of 10 pt (e.g., 1–3 pt = weak; 4–7 pt = moderate; 8–10 pt strong).

Finally, consensus statement and position stand papers were assessed through the 23 items of the AGREE II tool [42], grouped into six appraisal domains (e.g., (i) scope and purpose, (ii) stakeholder involvement, (iii) rigor of development, (iv) clarity of presentation, (v) applicability, and (vi) editorial independence), to be rated on a 1 (e.g., absence of information or concept is very poorly reported) to 7 (e.g., the quality of reporting is exceptional) points. To provide an overview of the quality of consensus statement and position stand papers included in the present integrative umbrella review, firstly the individual scores assigned to each item were summed to calculate a final composite score for each assessed paper. The total score could range from 161 (e.g., all items rated with 7 pts) to 23 (e.g., all items rated with 1 pt) pts, whereas 92 pts could be reached with an average 4 pt for each item. Hence, <92 pt, 92–115 pt, 116–138 pt, and 139–161 pt were considered suitable thresholds for low, average, good, or excellent quality, respectively.

Given the heterogeneity of study designs, appraisal tools were applied according to study typologies: (i) AMSTAR 2 for SLRs; (ii) JBI for ScRs; (iii) SANRA for narrative and conceptual manuscripts; and iv) AGREE II for consensus and position statements. Notably, the QATR tool was used as a complementary instrument to ensure cross-study comparability on general methodological criteria for SLRs and ScRs only. Furthermore, the integration of the appraisal of SLRs and ScRs using standard recommended tools (e.g., AMSTAR 2 and JBI, respectively) and a more generalist tool (e.g., QATR) was crucial to ensure that each appraised manuscript would not be over-penalized in relation to the study design and relative research area (e.g., social sciences, psychology, sport sciences). Finally, appraisal outcomes were not combined across tools, nor used as exclusion criteria. Instead, each tool informed the interpretation of evidence within its respective study typology. Discrepancies between tools were addressed qualitatively, acknowledging that each instrument evaluates different methodological dimensions. To ensure rigor and transparency in the appraisal process, each manuscript was independently assessed by at least two authors. Manuscripts were split among the coauthors in relation to the different study typologies (e.g., conceptual, NRs, positions: F.G., F.D.R, and C.R.; SLRs and ScRs: S.C., S.D., and L.C.). In case of disagreement between the two authors, a third author’s opinion was sought, with F.G. and S.C. acting as experts to solve potential discrepancies in the judgment. The outcomes of the appraisal process are included in the Supplementary Materials File S3.

### 2.6. Hierarchy of Evidence and Integrative Synthesis Logic

Given the heterogeneous nature of the included manuscripts (e.g., SLRs, ScRs, NRs, conceptual papers, and consensus and position papers), a hierarchical interpretive structure was applied during synthesis. SLRs and ScRs constituted the highest level of empirical evidence and primarily informed the identification of recurrent patterns and major trends in the support of elite athletes in relation to their mental health and career transitions (e.g., with a specific focus on the challenges and needs during career retirement). NRs contributed contextual and interpretive insights but were not treated as equivalent to systematic evidence. Consensus statements and position papers were classified as expert-derived sources and were used to identify normative recommendations, organizational priorities, and policy-level guidance. Finally, conceptual manuscripts provided theoretical scaffolding for interpreting empirical findings and for structuring the higher-order domains included in the final framework. Therefore, these evidence types were not weighted equally, with:

- SLRs and ScRs informing empirical convergence;
- Narrative, conceptual and expert opinion sources informing theoretical coherence and practical relevance.

This hierarchical structure ensured that the final proposed support framework was evidence-informed while appropriately distinguishing between empirical findings, conceptual propositions, and expert recommendations.

### 2.7. Data Extraction and Analysis

Contingent to manuscripts’ appraisal, six reviewers extracted the following information from the included articles: (i) bibliographic information (author(s), publication year, journal, DOI); (ii) geographical representation (e.g., country of the first author); (iii) general research setting information (e.g., dual career, mental health, career transitions, support services); (iv) specific methodological information (e.g., databases (n), references (n), type of study, scope of the study, examined variables); and (v) main outcomes in relation to the purpose of the present integrative umbrella review. From each included article, data were extracted independently by at least two authors, and in case of disagreement a third author’s opinion was sought. Data were synthesized narratively using a predefined extraction grid in an Excel file. To ensure a comprehensive overview of each included study, data extraction grid included all the selected variables and appraisal answers/scores. Data processing encompassed descriptive statistics in relation to the frequency of occurrence of recurrent themes and thematic analysis for clustering the collected information based on the identification of: (i) higher-order thematic areas, including symptom and risk domains (Matrix 1); (ii) higher-order intervention domains to ensure elite athletes’ adequate support in relation to their mental health and career transitions and assistance (Matrix 2); (iii) relevant intervention categories for possible implementation within an online platform (Matrix 3).

The thematic analysis and synthesis of the extracted data were independently performed by two authors for each included study, including six main phases: familiarization, coding, theme development, refinement, naming, and writing up [43]. In particular, the analytic process followed established procedures for qualitative evidence synthesis and matrix-based content analysis, drawing on methodological traditions of thematic analysis, framework synthesis, and cross-case matrix comparison [44,45,46]. During this phase, coders extracted all relevant segments referring to mental health symptoms, sport-specific stressors, career and identity processes, help-seeking barriers, protective factors, and recommended interventions. For Matrix 1, the two coders assessed the presence of core domains that could cover symptoms and risk factors, stressors, career and identity challenges, help-seeking barriers, and protective factors. Then, Matrix 2 captured recommended intervention domains commonly referenced in the literature. Finally, Matrix 3 expanded this structure to categories of possible implementation through digital support resources. To perform the coding task and document the presence/absence of retrieved domains and categories within included manuscripts, a dichotomous coding approach was applied (e.g., 1 = domain/intervention/category explicitly addressed; 0 = not addressed). To minimize potential bias in the process, after independent coding the two authors compared their matrices and discussed discrepancies, and in case of disagreement a third author’s opinion was sought. This multi-coder process aligns with best practice recommendations for enhancing reliability and reducing interpretive bias in qualitative research [47,48]. Notably, the thematic synthesis followed a hybrid analytic approach combining deductive and inductive elements. Deductive coding was informed by predefined domains derived from the PORTAL project objectives and our prior SLR [3], while inductive refinement allowed the identification of emergent concepts across heterogeneous study types. The analytic workflow included: (a) familiarization with all included manuscripts; (b) independent initial coding by two reviewers using structured extraction matrices; (c) development of cross-case matrices (Matrices 1, 2, and 3); (d) iterative theme generation and refinement; and (e) integration of themes into higher-order domains. Intercoder agreement was ensured through independent coding followed by systematic comparison of matrices. Discrepancies were discussed until consensus was reached, and a third reviewer adjudicated unresolved cases. Although no statistical coefficient was calculated (e.g., consistent with qualitative evidence synthesis standards [48]) the multi-step consensus process ensured analytic rigor. Coding and matrix development were conducted manually using structured Excel templates, which facilitated harmonized extraction across systematic reviews, conceptual papers, and consensus statements. The collection of this information (e.g., Matrix 1, 2, and 3) was crucial to structure a harmonized, evidence-based framework for possible implementation of a comprehensive set of services to support the mental health and career transitions of elite athletes through digital solutions (e.g., through an online platform, which represents the main objective of the PORTAL project). In particular, Matrices 1, 2, and 3 were designed as sequential analytic layers, which were: (i) Matrix 1 identifies needs and challenges; (ii) Matrix 2 translates these into higher-order intervention domains; and (iii) Matrix 3 operationalizes these domains into digital components. Based on this hierarchical logic, a final proposed framework was developed, graphically reflecting the convergence across these layers, and visually displaying the stepwise progression from evidence-derived needs to intervention domains and their corresponding digital implementation strategies, thereby ensuring that each digital feature is evidence-informed.

Finally, from all the included manuscripts, reference lists were also extracted to assess the extent of overlapping within the included studies (e.g., frequency counts). In particular, overlap analysis provides quantitative information on the repetition of primary studies across included reviews, crucial for ensuring evidence synthesis validity. Notably, although all instances of overlap were identified, not all repetitions exert the same influence on the evidence base. Studies appearing in only two included manuscripts typically contribute minimal bias and do not meaningfully shape review-level conclusions. Therefore, consistent with approaches used in previous umbrella reviews, we focused our interpretive analysis on studies cited in ≥4 included papers, representing substantial or high overlap. This threshold allowed us to distinguish trivial duplication from influential clustering. However, the Corrected Covered Area (CCA) index was also calculated using the formula proposed by Pieper et al. [49]. According to the literature [50], the following classification of the degree of overlap was considered: (i) 0–5% “slight overlap”; (ii) 6–10% “moderate overlap”; (iii) 11–15% “high overlap”; and (iv) >15% “very high overlap”.

## 3. Results

### 3.1. General Findings from the Integrative Umbrella Review

**Figure 1:**
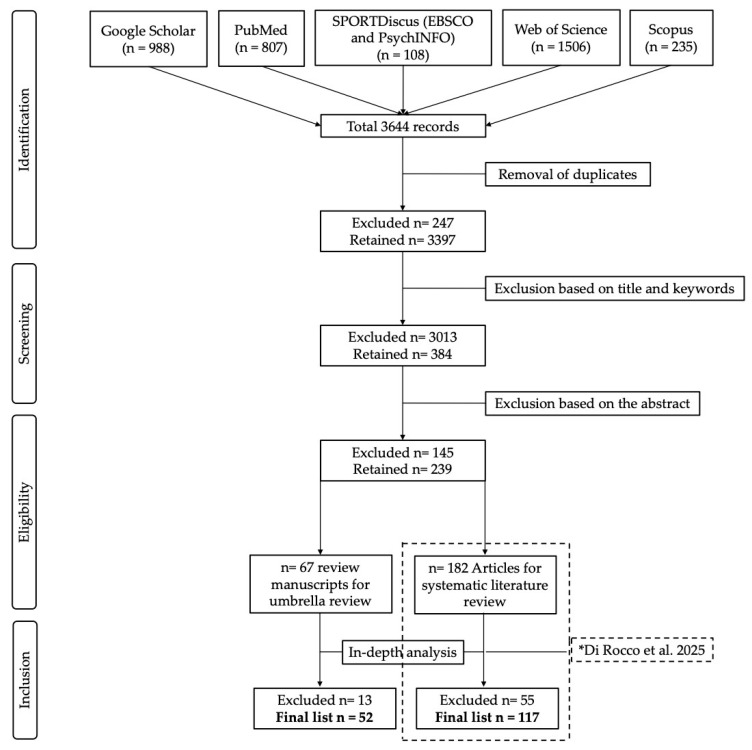
A flowchart representing the systematic process of review of the present study. * Manuscript published in Sports [3].

According to Di Rocco et al. [3], the initial search across selected databases returned 3644 records. After the multi-level screening and eligibility process, 67 review manuscripts were considered in the present study (Figure 1). Thirteen studies were excluded, as reported in Table 1. Hence, fifty-two manuscripts met the inclusion criteria and were included in the final list. Taken together, systematic and scoping reviews accounted for the most represented study category (46%). Studies were mostly conducted after the year 2020 (65%) in Europe (44%) followed by North America (27%), mostly in the field of mental health support (65%).

Overlap analysis was performed on a total of n = 2768 documented records (e.g., overall sum of reference lists of included manuscripts). Overlapping records resulted n = 830, with a total of n = 290 studies cited in multiple papers, distributed as follows: (i) n = 3 studies are among the references of >10 included manuscripts (dominant overlap); (ii) n = 6 studies are among the references of 7–9 included manuscripts (high overlap); (iii) n = 48 studies are among the references of 4–6 included manuscripts (moderate overlap); (iv) n = 60 studies are among the references of at least three included manuscripts (light overlap); (v) n = 173 studies are among the references of at least two included manuscripts (minimal overlap). The resulting CCA was 0.84%, indicating a slight overlap of primary studies referenced within included manuscripts. In this respect, the heterogeneous nature of the included sources and the broad thematic scope of the present study might have reduced the probability of multiple recurrent primary studies across the analyzed manuscripts. Furthermore, the substantial proportion of narrative, conceptual, and position papers (e.g., typically characterized by shorter and more selective reference lists) structurally lowers the likelihood of reference duplication, contributing to the low CCA value. Although this value suggests a low risk of duplication bias, overlap may still influence the synthesis. Indeed, several foundational studies on mental health prevalence, identity foreclosure, and retirement adjustment appeared repeatedly across multiple studies. To mitigate the influence of thematic dominance, we did not weight conclusions based on frequency of appearance, and coders monitored conceptual redundancy during thematic extraction. Overlap was therefore considered when interpreting convergent findings, particularly in domains where a small number of seminal studies informed multiple sources. The list of overlapping references is presented in Supplementary Materials File S2.

The final list of included studies is presented in Table 2, whereas Supplementary Materials File S3 includes the full set of collected information for each assessed manuscript (e.g., bibliographic information, specific methodological information, content information, outcome information, and appraisal results). Regarding the confidence of evidence, results of the appraisal process showed that:

i. The SANRA scores computed for NRs and conceptual manuscripts indicated an overall good quality of the evaluated studies (e.g., NRs: range = 6–10 pt/12; quality: n = 1 study strong; n = 5 studies moderate. Conceptual: range = 7–10 pt/10; quality: n = 13 studies strong; n = 2 studies moderate);
ii. The AGREE II scores calculated for consensus statement and position stand manuscripts revealed an average quality of guidelines of 106.1 ± 6.6 pt (range = 96–120 pt), with the lowest scores (range = 1–2.4 pt) assigned to item 7 (e.g., systematic methods were used to search for evidence), 8 (e.g., the criteria for selecting the evidence are clearly described), 14 (e.g., a procedure for updating the guideline is provided), 20 (e.g., the potential resource implications of applying the recommendations have been considered), and 21 (e.g., the guideline presents monitoring and/or auditing criteria). Conversely, the highest scores (range = 6.9–7 pt) were assigned to item 1 (e.g., the overall objective(s) of the guideline is (are) specifically described), 2 (e.g., the health question(s) covered by the guideline is (are) specifically described), 3 (e.g., the population (patients, public, etc.) to whom the guideline is meant to apply is specifically described) and 13 (e.g., the guideline has been externally reviewed by experts prior to its publication);
iii. For ScRs, the scores computed through the applied assessment instruments aligned (e.g., QATR: n = 5 studies with strong quality, score range = 7–8 pt; n = 1 study with moderate quality, score 4 pt. JBI: range of positive answers = 9–11 pt/11, indicating high quality for all considered studies);
iv. For SLRs, appraisal discrepancies emerged. Whilst the QATR scores denoted an overall good quality of the evaluated studies (n = 16 strong; n = 2 moderate), the AMSTAR 2 assessment highlighted that 78% of the included manuscripts fell into the Critically Low-to-Low quality categories (e.g., positive answers: 8.1 ± 2.0/16 items), whereas only one study presented High quality methodological standards [51]. Considering the discrepancy between the two assessment outcomes, it should be considered that QATR focuses on general methodological quality standards for the evaluation of a wide range of studies, whereas AMSTAR 2 was developed for systematic reviews of healthcare interventions and meta-analytic evidence for the rigorous appraisal and risk of bias evaluation of intervention studies [36]. In the scientific field of the present study, the empirical evidence might be weak in rigor and transparency, whereas conceptual literature predominates. Hence, the AMSTAR 2 results should be interpreted with caution in light of these domain-specific considerations. Notably, the predominance of “critically low” AMSTAR-2 ratings was not considered as reflecting the intrinsic quality of the included reviews but rather the limited suitability of AMSTAR-2 for evaluating review designs commonly used in sport sciences, psychology, and social science research [38]. Therefore, AMSTAR-2 results have confirmed that it systematically penalizes qualitative, conceptual, mixed-methods, and social science reviews due to non-applicable criteria [38]. For this reason, QATR scores were considered more appropriate for interpreting methodological quality in this field.

Although appraisal outcomes did not determine inclusion, they informed the interpretive weighting of evidence. Strong- to moderate-quality SLRs and ScRS (e.g., as assessed by QATR) contributed most strongly to empirical convergence, whereas narrative and conceptual manuscripts informed contextual and theoretical interpretation. Finally, consensus statements contributed expert-derived recommendations.

In general, the synthesis and presentation of outcomes can differ across reviews according to their specific objectives, with results typically presenting a summary of evidence with a specific focus on similarities and discrepancies in findings across the analyzed literature. To preserve epistemological clarity, the synthesis was structured into separate layers distinguishing: (a) theoretical insights from conceptual papers; (b) evidence-based findings derived from review-level manuscripts; and (c) expert-based recommendations from consensus and position statements. This stratified approach ensured that each form of evidence informed the present integrative review according to its methodological nature and intended purpose. In particular, conceptual manuscripts provide a general contextual background to introduce a detailed cross-review synthesis, specifically focused on challenges of providing adequate mental health and career transitions and assistance support to elite athletes, major intervention domains extracted from the analyzed studies, and possible intervention implementation through digital tools, such as an online platform. Finally, consensus and position manuscripts provide a necessary overview of expert opinions in the field and highlight major recommendations in the field of multidimensional support needs of elite athletes. Notably, the inclusion of heterogeneous study designs introduces variability in evidentiary strength. While this integrative approach is appropriate for mapping complex, multi-level determinants of athlete mental health and career transitions, conceptual and opinion-based sources were not weighted as empirical evidence, limiting their contribution to a contextual theoretical preliminary overview and major recommendation trends in the field, respectively.

**Table 2:** A list of the included studies.

Code	Year	Authors	Typology	Code	Year	Authors	Typology
[52]	2015	Schinke et al.	Conceptual	[53]	2021	Wendling and Sagas	Conceptual
[4]	2016	Knights et al.	Systematic review	[54]	2022	Chen and Bansal	Conceptual
[55]	2016	Rice et al.	Systematic review	[56]	2022	Chroni and Dieffenbach	Conceptual
[57]	2016	Stambulova	Conceptual	[58]	2022	Colagrai et al.	Scoping review
[59]	2017	Schinke et al.	Position Stand	[60]	2022	Delfin et al.	Systematic review
[61]	2018	Mannes et al.	Systematic review	[62]	2022	Purcell et al.	Conceptual
[63]	2018	Moesch et al.	Position Statement	[64]	2023	Assa & Reizer	Systematic review
[65]	2018	Souter et al.	Narrative Review	[66]	2023	Crossen et al.	Systematic review and meta-study
[67]	2019	Buckley et al.	Systematic review	[68]	2023	Nuetzel	Systematic review
[69]	2019	Castaldelli-Maia et al.	Systematic review	[70]	2023	Pena-Pérez & Portela-Pino	Systematic review
[71]	2019	Gouttebarge et al.	Systematic review and meta-analysis	[72]	2023	Rebelo et al.	Scoping review
[73]	2019	Stillman et al.	Narrative Review	[74]	2023	Voorheis et al.	Scoping review
[75]	2019	Swartz et al.	Narrative Review	[76]	2023	Waud and Weese	Narrative Review
[77]	2019	Van Slingerland et al.	Position Statement	[78]	2024	de Oliveira Camilo et al.	Meta-Ethnographic Synthesis
[79]	2020	Giles et al.	Narrative Review	[80]	2024	Fatt et al.	Umbrella review
[81]	2020	Gorczynski et al.	Conceptual	[82]	2024	Fatt MClinPsy et al.	Systematic review
[21]	2020	Henriksen et al.	Consensus Statement	[83]	2024	Gill et al.	Systematic review
[84]	2020	Küttel & Larsen	Scoping review	[85]	2024	Gorczynski et al.	Narrative Review
[86]	2021	Barth et al.	Systematic review	[87]	2024	Kussman and Choo	Conceptual
[88]	2021	Ekengren et al.	Conceptual	[89]	2024	Prior et al.	Scoping review
[90]	2021	Larsen et al.	Conceptual	[51]	2024	Runacres & Marshall	Systematic review and meta-analysis
[91]	2021	Perry et al.	Scoping review	[92]	2024	Schinke et al.	Position Stand (revised/updated)
[93]	2021	Poucher et al.	Conceptual	[1]	2024	Stambulova et al.	Position Statement
[94]	2021	Si et al.	Conceptual	[95]	2024	Stevens et al.	Conceptual
[96]	2021	Stambulova et al.	Position Stand (revised/updated)	[97]	2024	Wylleman	Conceptual
[98]	2021	Vella et al.	Systematic review and meta-synthesis	[99]	2025	Li et al.	Conceptual

### 3.2. Synthesis of Included Manuscripts in Relation to Study Typology

The following narrative synthesis is intentionally descriptive to ensure transparency and traceability across heterogeneous evidence types. Then, the primary analytical contribution of this integrative umbrella review is represented by Matrices 1, 2, and 3, which consolidate cross-review convergence and/or divergence, methodological consistency, and higher-order thematic integration.

#### 3.2.1. Overview of Theoretical Insights Derived from Included Conceptual Manuscripts

Mental health in elite sport is best understood as a lifespan developmental process, shaped by predictable and unpredictable transitions that require anticipatory, phase-specific scaffolding. In particular, Ekengren et al. [88] presented the developed and validated applied framework for career-long psychological support services in the context of Swedish handball, an efficient career assistance practice complemented by applied experiences. Similarly, Wylleman [97] provided an overview of some of the recent developments in the application of sport psychology at the Olympic level, with a specific focus on the relationship between mental performance and mental health. Both works provide substantial insight into the need for a developmental framework for assisting mental health and career support in elite sport, showing that athletes’ needs evolve across initiation, mastery, and discontinuation, and that transitions constitute periods of heightened vulnerability. In fact, whether normative (e.g., junior-to-senior), non-normative (e.g., injury), or quasi-normative (e.g., Olympic cycles), changes and challenges accompanying career transitions need tailored interventions and assistance to effectively meet athletes’ needs. Their work aligns directly with previous research conducted by Stambulova’s [57] on the holistic lifespan and cultural praxis paradigm, which reconceptualized athletes as whole persons whose athletic, psychological, psychosocial, academic/vocational, physical, and financial domains develop in parallel. Stambulova’s career development and transition model [57] provides the structural logic that underpins the entire field: transitions are not events but multi-phase processes, and effective support requires integrating career development, transition management, and career assistance interventions within culturally attuned, ecologically valid systems. This developmental foundation is operationalized by the study of Schinke et al. [52], mapping Olympic preparation as a sequence of meta-transitions and the psychological support provided in the context of the Canadian Olympic Men’s Boxing Team. In particular, the study emphasized how each meta-transition is characterized by distinct demands, resources, and coping requirements, urging the establish-ment of ecologically grounded support systems and longitudinal psychological assistance that are culturally embedded and responsive to athletes’ lived contexts. This ecological logic is echoed in the work of Larsen et al. [90], offering a comprehensive overview of knowledge and strategies most suitable for mental health promotion and prevention in elite sport, with a broad focus on how interventions can be structured and implemented, including actions to be taken when challenges become illnesses and when diagnosed mental disorders require adequate and timely treatment. Their work also showed that global disparities in service provision, organizational cultures, and governance structures still shape athletes’ access to care and their willingness to seek help. Together, these studies reveal that mental health cannot be separated from the systems and environments in which athletes train, compete, and transition.

The identity dimension emerged as another major pillar within the included studies. Wendling and Sagas [53] conceptualized retirement as a liminal phase marked by identity crisis, emotional turbulence, and temporary moratorium. Their model showed that identity reconstruction is essential for achieving post-sport identity synthesis. This identity-focused perspective aligns with the study of Chen and Bansal [54], reporting that injury-induced, unplanned career termination triggers profound identity disruption, emotional trauma, and vocational uncertainty, requiring structured, theory-informed counseling to support adaptive coping and re-engagement. The study by Stevens et al. [95] further expanded by proposing a novel perspective for tackling mental health challenges through a social identity approach, demonstrating that social identities (e.g., sense of belonging, shared meaning, group-based support, and identity-aligned leadership) function as core psychological resources that buffer stress, enhance resilience, and shape athletes’ capacity to navigate transitions. In particular, their work bridges individual identity processes with team-level and organizational dynamics, showing that identity is not only personal but relational and collective. Further, Chroni and Dieffenbach [56] introduced a role-specific transition that has been largely overlooked: the elite athlete-to-coach shift. Their work revealed that this within-sport transition involves unique identity, relational, and competence-related challenges, including the need to renegotiate self-concept, develop new interpersonal and pedagogical skills, and avoid perpetuating harmful norms internalized during one’s athletic career. Their findings resonate strongly with Wendling and Sagas’ liminality model [53], Stevens et al.’s social identity framework [95], and Stambulova’s cultural praxis paradigm [57], showing that the athlete-to-coach transition is not merely a vocational shift but a deep identity transformation that requires structured psychological support, reflective practice, and organizational scaffolding.

The cultural and structural context is another major emerged thematic field. Here, two studies conducted in the Chinese context provide a powerful illustration of how national sport systems shape mental health trajectories. In particular, Si et al. [94] showed that mental health management must be adapted to the structural realities of the Chinese Whole Nation System, where athletes train in centralized, state-run environments. Their sport-center-based framework, which combines annual two-dimensional screening, follow-up and emergency interventions, and mental health literacy education, demonstrated how large-scale infrastructures can institutionalize prevention and early detection. Further, Li et al. [99] deepened this contextualization by presenting an athlete-centered, reflective career assistance intervention tailored to the same system. Their use of individual, group, and meta-reflection, culminating in polyphonic reflective tales, showed how reflective practice can enhance autonomy, coping, and identity work in environments where athletes often have limited agency. These studies exemplify Stambulova’s cultural praxis paradigm [57], which argues that career research and interventions must be culturally grounded, contextually aware, and reflexive.

The literacy, competence, and organizational leadership dimension also represents a major area of intervention. Gorczynski et al. [81] emphasized mental health literacy and cultural competence as foundational competencies for athletes, coaches, and organizations, arguing that effective prevention and early intervention depend on context-specific, culturally attuned education. Purcell et al. [62] translated these insights into an organizational framework, embedding mental health within system-level structures, considering the relationships between an individual athlete and the broader social and cultural contexts. The study provided actionable recommendations that sporting organizations can adopt and tailor to meet their unique needs for stepped care pathways and routine monitoring. Their study also called on major sporting organizations to expand their policies toward the provision of adequate mental health services for improving athletes’ well-being and their psychological safety within the elite sport context. Also, Stevens et al.’s study [95] reported that identity-based leadership can actively foster healthier climates and more resilient athletes. Relative to problematic intersections between mental health and career transitions, Kussman and Choo [87] showed that disordered eating and eating disorders cluster around transitional phases and require multidisciplinary detection and treatment, whereas Poucher et al. [93] documented how stigma, gendered norms, and mental-toughness cultures suppress disclosure and delay care. These findings reinforce the need for psychologically safe environments, early-intervention pathways, and culturally competent clinical support. Hence, literacy, organizational cultures, and leadership are not peripheral but central mechanisms through which systems shape the mental health of elite athletes.

Overall, these manuscripts converge on a model in which effective athlete mental health and career transition support should be anticipatory, continuous, culturally competent, relationally grounded, reflective, clinically responsive, and ecosystem-level. The findings highlight that well-being is shaped by the interplay of developmental trajectories, identity processes, social identities, cultural contexts, organizational structures, and role-specific transitions. They also demonstrate that interventions must integrate literacy and competence development, identity- and group-based mechanisms, organizational accountability, multidisciplinary coordination, and sustained follow-up beyond retirement, while addressing high-risk clinical conditions and the cultural, structural, and social identity factors that shape vulnerability, help-seeking, and well-being across the athletic lifespan.

#### 3.2.2. Cross-Review Synthesis

Across the analyzed 30 reviews (e.g., n = 6 NRs; n = 6 ScRs; n = 18 SLRs), a comprehensive, multidimensional, culturally grounded, and increasingly methodologically reflexive understanding of elite athletes’ mental health emerged. Rice et al. [55] provided a foundational synthesis showing that elite athletes experience comparable rates of common mental disorders to the general population, while facing elevated risk in contexts such as injury, overtraining, performance decline, and transitions into and out of sport. These early insights were further substantiated by epidemiological findings from Gouttebarge et al. [71], Runacres and Marshall [51], and Mannes et al. [61], who documented high prevalence of distress, sleep disturbance, and anxiety/depression among both current and former athletes. Transition-focused reviews by Barth et al. [86] and Knights et al. [4] further highlight the psychological vulnerability associated with retirement, identity disruption, and loss of structure.

The need for coherent, high-quality mental health policy is further underscored by Vella et al. [98], providing a meta-synthesis of thirteen formally endorsed mental health position statements revealing substantial variability in scope, quality, and methodological rigor. Furthermore, their work highlighted that stakeholder involvement is often limited, development processes lack transparency, and applicability guidance is weak. Their findings reinforced the broader literature’s concerns about fragmented service provision and inconsistent organizational responsibility, and emphasized the need for unified, evidence-based, contextually grounded guidelines. In this respect, digital platforms could standardize mental health plans, operationalize guidelines, and support monitoring, referral, and literacy.

Career transition challenges and needs emerged as a central vulnerability in the elite sport context. In particular, Waud & Weese [76] showed that retirement is rarely a neutral and/or smooth process. In fact, athletes frequently experience identity loss, emotional distress, and difficulties in reconstructing their identity and purposes beyond sport. Whilst a strong athletic identity enhances performance during competitive years, it increases vulnerability when the athlete role is lost. Their recommendations, including career planning, identity diversification, mental health support, and structured transition programs, highlighted the need to establish athlete-centered and lifespan-oriented support systems.

Buckley et al. [67] added a crucial dimension to career retirement challenges by demonstrating how abrupt career termination affects the relationship between food, body, and identity in elite athletes. Their concept of “Athletic Body Transition” showed that body dissatisfaction, body grief, and compensatory behaviors often intensify in early retirement, especially among athletes with strong athletic identity or histories of weight-controlled sports. These maladaptive patterns reflect the long-term influence of sporting subcultures that valorize leanness, discipline, and body monitoring. Similarly, Voorheis et al. [74] showed that high athletic identity, abrupt retirement, and financial insecurity hinder athletes’ transition out of sport, whereas a healthy sport–life balance, gradual disengagement, and personal control over retirement ease the necessary adjustments. In fact, retirement often brings identity loss, reduced social networks, limited career direction, and risks to physical and mental health, determining the need for stepwise programming that helps athletes make sense of their careers, develop a broader identity, build agency through planning and new routines, and normalize retirement as an ongoing process.

The review of Gorczynski et al. [85] on loneliness and emotional blunting expanded the symptom landscape beyond traditional mental health diagnostic categories. Loneliness may be highly prevalent during injury and retirement and is strongly associated with depression, anxiety, suicidality, and substance use. Furthermore, it may be exacerbated by disrupted social networks, stress, and high athletic identity. However, emotional blunting, often linked to antidepressant use, may further impair social functioning, motivation, and interpersonal connection, yet remains unstudied in elite athletes. Pena-Pérez & Portela-Pino [70] reinforced that mental health, physical health, and quality of life are deeply intertwined during and after sport careers, with injuries, pain, sleep disturbance, alcohol consumption, and nutritional habits shaping post-career well-being. Further, Stillman et al. [73] provided the most detailed synthesis of psychotherapeutic approaches in mental health symptoms and disorders in elite athletes, emphasizing that psychotherapy alone or combined with pharmacological strategies is central to managing mental illness symptoms in elite athletes. They highlighted diagnostic complexities, stigma, confidentiality concerns, and the need for adapted interventions and family-based approaches. Colagrai et al. [58] and other authors argued for a dimensional perspective on mental health, conceptualizing health and illness as coexisting continua ranging from reduced to effective functioning, reinforcing the need for psychological support aimed at both maintenance and enhancement of mental health. De Oliveira Camilo et al. [78] further showed that depression, anxiety, mania, and suicidal ideation may be triggered by performance pressure, injuries, normalized violence, harassment, discrimination, and abuse in high-performance sport, with athletes often skilled at hiding emotional pain.

Küttel & Larsen [84] strengthen this environmental and contextual perspective by mapping risk and protective factors for mental health in elite sport. Injury, overtraining, adverse life events, perfectionism, identity foreclosure, career dissatisfaction, poor sleep, maladaptive coping, and stigma are among the most prevalent risk factors, while autonomy, positive relationships, mastery-oriented climates, access to support, recovery, and basic needs satisfaction function as protective factors. Hence, a great emphasis on the need of person–environment balance was highlighted. Furthermore, Fatt et al. [80] and Fatt McPlinsy et al. [82] showed that life changes such as injuries, performance drops, coaching changes, off-season transitions, pregnancy, puberty, body composition changes, upcoming competitions, and career retirement can destabilize this balance, often leading to disordered eating behaviors that persist or shift post-retirement. In this framework, help-seeking remains rare, with risk and protective factors shaped by individual, sociocultural, and sport-specific pressures. Gendered patterns further shape mental health outcomes, as reported by Souter et al. [65] highlighting that male athletes may face intersecting risks (e.g., injury, overtraining, performance pressure, disordered eating, substance use, and stigma) exacerbated by norms discouraging emotional expression and help-seeking. Perry et al. [91] complemented this concept by demonstrating that elite female athletes face distinct vulnerabilities, mostly as higher prevalence of anxiety, depression, and disordered eating, sport-specific pressures related to leanness and appearance, and sociocultural stressors including gender stereotypes, sexualization, unequal opportunities, and limited financial support. Nuetzel [68] also added that female athletes tend to appraise stressors more negatively, experience stronger tension and worry, and rely more on emotion-focused coping, whereas male athletes more often use active coping patterns shaped by gender role socialization. Finally, Prior et al. [89] argued that limited cohesion between research and applied practice leaves practitioners without clear guidance for designing targeted interventions. Although eating disorders are more prevalent among women, framing them as a “female issue” reinforces stigma and discourages disclosure among male athletes, highlighting the need for gender-inclusive interventions.

Measurement of mental health and disorders emerged as a major methodological gap and uneven domain. Giles et al. [79] argued that well-being is inconsistently defined and poorly measured, with sport research dominated by proxy indicators rather than multidimensional constructs. Furthermore, Rebelo et al. [72] confirmed that well-being monitoring in elite sport focuses almost exclusively on fatigue, sleep, soreness, and mood, neglecting psychological, social, and eudaimonic dimensions. Colagrai et al. [58] also called for instruments that encompass both protective factors and symptom screening. In this respect, Nuetzel [68] emphasized that mental health literacy is central from a young age in educating athletes in relation to relevant mental skills to successfully cope with stressors. Furthermore, educating coaches and teammates about social support may reduce anxiety and depressive symptoms.

Inter-relationships between stressors, appraisal, coping strategies, and mental health outcomes remain unclear, with intervention evidence remaining limited. Delfin et al. [60] showed that only nine sport-specific mental health interventions for elite athletes have been evaluated in the past decade, with small samples, inconsistent outcome measures, limited theoretical grounding, and variable methodological quality. Interventions combining psychoeducation, mindfulness, skills training, and group discussion showed promising outcomes by improving knowledge, reducing stigma, and lowering stress, but evidence for symptom reduction is sparse. Standardized, sport-specific mental health reporting should be prioritized to ensure consistency and contextual relevance. Effective support also requires examining the roles of key stakeholders and reducing over-reliance on sport psychologists by upskilling coaches and support staff to provide everyday mental health support [89]. Notably, although studies vary in timing relative to the COVID-19 pandemic, its global impact on athletes’ mental health warrants further exploration. Assa & Reizer [64] highlighted the importance of online psychological support, especially during periods of crisis (e.g., such as COVID-19, injury or retirement), recommending that organizations fund access to sport psychology practitioners to enhance resilience and well-being. In worse scenarios, poorly supported transitions, loneliness, and emotional blunting might also lead to suicidal ideation, attempts, and mortality, especially in elevated risk specific subgroups of former athletes [83]. Furthermore, Castaldelli-Maia et al. [69] identified stigma, low mental health literacy, hypermasculinity, negative past experiences, and busy schedules as major barriers to help-seeking. Cultural factors including gender norms, racialized expectations, religious non-disclosure, and economic dependence shape vulnerability and access. In this context, coaches play a pivotal role in shaping attitudes toward treatment, and brief anti-stigma interventions might elicit promising outcomes in enhancing mental health in elite sport. Notably, when considering how to best support athletes’ mental health, the roles of various stakeholders should be examined. Moving away from reliance on a sport psychologist through the upskilling of coaches and sport staff might facilitate a more collaborative approach to athlete mental health support.

Paralympic athletes face additional, disability-specific stressors, as reported by Swartz et al. [75], showing that chronic pain, misclassification, inaccessible environments, and trauma histories compound vulnerability, while stereotypes and research gaps obscure their needs. Furthermore, Crossen et al. [66] revealed that identity construction in disability sport is shaped by limited narrative options. While many athletes describe disability as empowering their sporting success, they often align with able-bodied ideals, masking broader societal narratives that resurface during retirement or employment challenges. Sport may shield disabled athletes from everyday barriers, making the transition from “disabled athlete” to “disabled person” particularly difficult. Hence, the review argued that elite disabled athletes should be recognized as educational figures whose experiences can inform better support and understanding. Furthermore, when addressing mental health and career transition service provision, athletes with disabilities need a tailored support approach. Across most included studies, methodological limitations emerged as a major issue, with over-reliance on cross-sectional designs, small samples, inconsistent definitions of “elite athlete,” limited diagnostic interviews, and a near absence of intervention studies. A convergence emerged when considering athlete mental health as a lifespan, multidimensional, culturally embedded, relationally mediated, and structurally shaped phenomenon. Identity, embodiment, organizational culture, stress exposure, coping repertoires, gendered and racialized dynamics, disability-specific stressors, loneliness, emotional blunting, and the quality of transitions all shape mental health and effective career transition outcomes. Collectively, the evidence points toward the need for integrated, longitudinal, theoretically grounded, and athlete-centered mental health systems capable of supporting athletes through distress, disorder, and toward sustained flourishing across their careers and into retirement. Digital platforms may offer a promising implementation pathway by delivering confidential psychoeducation, transition planning, body-change guidance, stigma-reducing content, culturally tailored resources, symptom monitoring, and rapid referral pathways, addressing the accessibility, literacy, and structural barriers repeatedly identified across the literature.

#### 3.2.3. Consensus/Position Papers Overview

Across the seven position and consensus papers, a coherent set of recommendations emerged that reframe elite athlete mental health and career development as inseparable from the structural, cultural, and technological systems that surround athletes throughout their careers. Schinke et al. [59] established that mental health exists on a continuum and that subclinical levels of psychological distress determine a negative impact on individual functioning, underscoring the need for early detection and continuous monitoring. In this respect, digital platforms could be uniquely positioned to deliver support services through routine screening, symptom-tracking, and automated alerts. The 2024 ISSP position stand [92] further defined mental health as “the dynamic interaction of our psychological, social, and emotional well-being,” calling for systems capable of capturing fluctuations across multiple continua (e.g., cognitive, emotional, behavioral, interpersonal). In this framework, digital infrastructures can integrate multidimensional data streams and provide personalized feedback.

Henriksen et al. [21] emphasized that mental health is a core component of a culture of excellence, and that environments can nourish or malnourish athlete mental health, recommending coordinated systems of care, mental health officers, and organization-wide literacy initiatives. These recommendations translate directly into digital solutions such as centralized case-management dashboards, cross-team communication channels, and online literacy modules accessible to all stakeholders. Furthermore, Moesch et al. [63] highlighted the fragmentation of service provision across Europe, noting that competencies, certification issues, and professional boundaries differ strongly across European countries, negatively impacting early screening and interventions. Digital platforms could mitigate these structural inconsistencies by offering standardized assessment pathways, tele-referral systems, and secure communication between sport psychologists, clinicians, and medical staff. Van Slingerland et al. [77] further demonstrated that stigma, lack of specialized practitioners, and system-level barriers deter help-seeking, observing that athletes often compete in cultures that stigmatize mental health, discouraging help-seeking, and that many “choose to suffer in silence because they are concerned that practitioners will not understand their unique needs.” Their Canadian model explicitly recommended integrated, sport-specific mental health care systems. These recommendations can be operationalized through digital infrastructures offering confidential access points, anonymous self-screening, and remote consultations with clinicians trained in sport-specific contexts.

Career transitions, especially retirement, were consistently identified as high-risk periods requiring structured, anticipatory, and longitudinal support. Stambulova et al. [96] conceptualized athletes as whole persons navigating interdependent athletic, psychological, psychosocial, academic-vocational, financial, and legal layers, with crisis-transitions marked by a “decrease in self-esteem, lasting emotional discomfort, and disorientation in decision-making.” The FEPSAC Dual Career Position Statement [1] extended this concept by showing that dual-career pathways are shaped by context, transitions, resources, and support systems. Both papers recommended structured planning, identity development, and empowerment-oriented environments, recommendations that could be implemented through digital support services that can translate into personalized transition roadmaps, interactive identity exploration modules, literacy, and integrated academic-vocational planning tools. The 2024 ISSP position stand further noted that career termination is a particularly challenging transition that could trigger pre-existing and previously unrecognized issues, while Moesch et al. [63] showed that athletes considering or planning retirement might present higher rates of depressive symptoms, reinforcing the need for digital systems capable of longitudinal follow-up, relapse prevention prompts, and remote access to psychological support during and after retirement.

Across all the manuscripts, a shared set of recommendations emerged: (1) continuous mental health monitoring through validated digital screening tools, symptom-tracking, and automated alerts; (2) integrated care coordination, where digital platforms could centralize communication among the relevant stakeholders (e.g., mental health officers, sport psychologists, clinicians, coaches, and dual-career support providers); (3) mental health literacy and stigma reduction through online education modules, athlete-led digital testimonials, and culturally adaptable resources; (4) dual-career and transition planning through digital career-mapping tools, academic-vocational integration, and structured retirement transition pathways; (5) culturally sensitive, cross-national accessibility, ensuring that athletes across countries and systems can access consistent, evidence-based support regardless of local service provision; (6) confidential access points enabling athletes to seek help anonymously, reducing stigma and fear of repercussions; and (7) longitudinal follow-up, where digital systems could maintain continuity of care across teams, federations, and post-sport life. Collectively, the manuscripts highlighted that it is essential to operationalize modern, evidence-informed mental health and dual-career support systems, enabling scalable, personalized, and culturally adaptable care across the entire athletic lifespan, including the critical transition into retirement.

### 3.3. Development of a Harmonized Framework for Mental Health and Career Transition Support Through Digital Intervention Tools

The thematic analysis performed on extracted data determined the identification of higher-order thematic areas, higher-order intervention domains, and relevant intervention categories for possible implementation within an online platform for the development of a harmonized, evidence-based framework to ensure elite athletes’ adequate support in relation to their mental health and career transitions. To ensure full operationalization of the analytical outputs, three matrices were designed as sequential layers of synthesis: Matrix 1 identifies main thematic areas; Matrix 2 translates these domains into higher-order intervention areas; and Matrix 3 maps these interventions onto digital implementation categories. This hierarchical structure informed the development of the final integrated framework.

Matrix 1 provides the first level of analytical integration by mapping convergent and divergent symptom domains, sport-specific stressors, and help-seeking barriers across the included studies. This matrix synthesizes patterns that are not visible when studies are examined individually. Regarding the identified higher-order thematic areas (Table 3), five main thematic domains emerged:

1. Symptoms and Risk Factors: This domain captures several mental-health-related aspects (e.g., anxiety, depression, distress, sleep problems, disordered eating/relative energy deficiency (RED), substance use, trauma, pain, concussion, emotional blunting, gambling, loneliness) reported in almost all the included studies. In fact, across the 52 included studies, mental health in elite sport is consistently conceptualized as a dynamic, career-embedded construct shaped by the interaction of symptoms, sport-specific stressors, career transitions, help-seeking barriers, and protective factors. Symptoms and risk factors were the most frequently represented domain, with widespread evidence of psychological distress across multiple career stages. These symptoms often intensified during periods of uncertainty such as injury, deselection, or retirement.
2. Sport Specific Stressors: This is a core domain across the dataset (e.g., including injury, overtraining, load imbalance, performance pressure, classification issues (para sport), travel, competition cycles, organizational constraints, maltreatment, abusive coaching) and represented in most included studies. Furthermore, sport-related stressors often intensify in late-career phases marked by declining performance or reduced support.
3. Career Transitions and Identity: This domain was extremely represented in the dataset, including retirement, injury-related transitions, identity loss, liminality, athlete-to-coach transition, dual career, Olympic cycle transitions, cultural transitions (moving abroad, training centers), and retirement difficulties. In particular, career-related challenges, particularly retirement and dual-career transitions, emerged as a central vulnerability. Studies highlighted emotional, identity-related, and practical demands associated with balancing sport and education/work and preparing for life after sport. Dual-career research showed that inadequate educational or vocational preparation exacerbates distress, identity loss, and reduced well-being, reinforcing the need for dual-career programs that support adaptability, employability, and identity diversification.
4. Help-Seeking Barriers and Cultural Factors: This domain is also strongly represented. It includes stigma, gendered norms, cultural norms, racialized/gendered barriers, access issues, literacy gaps, confidentiality concerns. Furthermore, barriers to help-seeking often worsen during retirement when support structures diminish. The consideration of these aspects is essential when designing, structuring and implementing an online support platform.
5. Protective Factors and Support Systems: This domain includes several social and individual resources for mental health and career transition management (e.g., social support, resilience, coping skills, organizational responsibility, psychologically safe environments, multidisciplinary teams, identity resources, sense of belonging and group identity), which are presented in many included studies. This domain highlights that supportive environments, high-quality development settings, strong social networks, resilience resources, and athlete-centered organizational cultures can nourish mental health across the entire career, especially during retirement. However, coherent values, coordinated systems, and explicit attention to identity development and life-after-sport planning are necessary elements for successful mental health promotion and career development.

Overall, strong convergence emerged across the included studies regarding psychological distress during injury, deselection, and retirement, while evidence on cultural and gender-specific differences remained inconsistent. Methodological heterogeneity was high, but symptom domains showed substantial overlap.

Regarding intervention areas and strategies for athletes’ mental health and career transition support, a strong consensus emerged that effective support in elite sport requires multi-level, career-long interventions, with particular emphasis on preparing athletes for, and supporting them through, the transition out of sport (Table 4). In particular, Matrix 2 advances the analytical synthesis by identifying higher-order intervention domains consistently recommended across included studies, highlighting areas of consensus, gaps, and methodological variability in proposed support strategies. Ten interconnected domains were identified:

1. Screening and Early Detection: This domain was widely identified as foundational, with strong consensus on the need for systematic detection of psychological distress, risk factors, and contextual vulnerabilities across the athletic career, urging the need for sport-specific, developmentally sensitive screening systems.
2. Psychoeducation and Literacy: Educational interventions were similarly prominent, emphasizing mental health literacy, stigma reduction, and dual-career competence for athletes, coaches, parents, and support providers.
3. Self-Management Skills: Psychological skills training including coping, resilience, emotional regulation, and career adaptability was widely recommended to help athletes manage performance and career demands.
4. Lifestyle and Load Management: This domain includes interventions focused on preventing overload and burnout, particularly during periods of high academic, vocational, or competitive demand, and called for coordinated planning across sport and education/work settings.
5. Clinical Treatment Pathways: This domain, including referral systems, multidisciplinary care, and mental health officer roles, was identified as essential for continuity of care, especially during transitions and retirement.
6. Crisis and Risk Management: Crisis and safeguarding interventions should address abuse, excessive weight control, and organizational toxicity.
7. Social Support and Peer Programs: Support systems (e.g., coaches, peers, families, mental health practitioners, and dual-career providers) were consistently described as central to well-being.
8. Identity, Transition and Career Support: Identity and transition interventions were especially prominent, reflecting widespread recognition that retirement requires structured support, identity diversification, and long-term planning.
9. Organizational Policy and Environment: Policy and governance interventions emphasized coherent systems and integrated dual-career frameworks.
10. Cultural Safety and Inclusion: Cultural inclusion cut across all domains, underscoring the need for context-sensitive, culturally responsive approaches.

Overall, the intervention domains show clear agreement on the need for screening, monitoring, literacy, and transition planning, whereas divergence persists regarding the timing, intensity, and delivery models of support. Notably, few studies addressed cross-national implementation challenges.

Regarding possible implementation within an online platform for the development of a harmonized, evidence-based framework, Matrix 3 translates the cross-study evidence into implementation-ready categories, enabling comparison of digital support strategies and revealing structural gaps in current service provision. This matrix operationalizes the umbrella-level integration central to the aims of the present study and of the broader PORTAL project. In particular, 14 main intervention categories were identified (Table 5): (1) mental health symptom screening; (2) RED-S/eating disorder screening; (3) concussion/trauma psychological screening; (4) training load/stress monitoring; (5) mental health literacy; (6) stigma reduction and help-seeking literacy; (7) cultural safety and inclusion literacy; (8) coping skills; (9) emotion regulation and mindfulness; (10) resilience and stress-management skills; (11) peer support/team-based support; (12) coach education and supportive communication; (13) family involvement modules; and (14) identity, transition and dual-career support. Overall, digital implementation strategies showed the highest cross-study convergence, with strong support for scalable tools. However, gaps remain in culturally sensitive design, long-term follow-up, and integration with existing sport structures.

Across the included studies, platform-relevant intervention categories suggest that digital systems might address the full continuum of athletes’ mental health and career development needs. Screening was consistently prioritized, with strong support for the early identification of psychological distress, emotional dysregulation, and other indicators of compromised well-being [4,55,71,75,80,83]. Although fewer studies examined screening for RED-S or concussion-related psychological effects, mental health screening was emphasized across empirical work and position statements [1,21]. These findings highlight the value of integrated, adaptive digital tools capable of detecting early distress and flagging risk during vulnerable periods, particularly the transition out of sport. The monitoring of training load, stress, and contextual pressures was another prominent category, with several studies documenting the cumulative burden of high training demands, academic responsibilities, and dual-career pressures [72,84,95,98]. Digital platforms can integrate physiological, behavioral, and self-reported indicators to detect declines in well-being, especially as athletes approach retirement. Education and literacy were widely represented, emphasizing mental health literacy, stigma reduction, and culturally responsive education for athletes and support providers [60,61,69,70,91]. Position statements stressed the need for education that also supports career adaptability and identity development [1,21]. Digital platforms can operationalize this through modular, personalized learning pathways. Self-management skills including cognitive behavioral strategies, emotional regulation, resilience, and coping were consistently endorsed [58,73,74,76,79,89]. These skills are especially critical during dual-career and retirement transitions [56,57], and digital delivery enables adaptive, interactive reinforcement. Support system interventions (e.g., peer, coach, and family support) were also central [53,59,77,88,93]. Digital platforms could strengthen these networks through shared resources and structured involvement during retirement planning. Finally, identity and career development support emerged as critical, with strong emphasis on identity diversification, employability, and structured transition planning [56,72,89,96]. In this context, digital systems must explicitly support long-term career trajectories.

Overall, this body of information suggests that digital platforms might be uniquely positioned to deliver evidence-based, comprehensive, developmentally informed, career-long support, especially during the transition out of sport. However, although several included manuscripts highlighted the potential of digital and hybrid support systems, empirical evidence on the effectiveness of digital interventions specifically targeting elite athletes remains limited. For this reason, the digital implementation categories identified in Matrix 3 should be interpreted as evidence-informed opportunities rather than empirically validated solutions.

To enhance analytical transparency, the three matrices were subsequently integrated into a structured mapping process presented in Table 6. In particular, Matrix 1 identifies the core needs and challenges emerging from the included evidence. Matrix 2 translates these needs into higher-order intervention domains. Finally, Matrix 3 operationalizes these domains into potential digital implementation components. Hence, Table 6 synthesizes this hierarchical progression, showing how each proposed digital component is directly anchored to specific needs and intervention domains. This mapping clarifies the analytic pathway from evidence extraction to potential framework construction, toward the possible development of a tailored digital support model.

**Figure 2:**
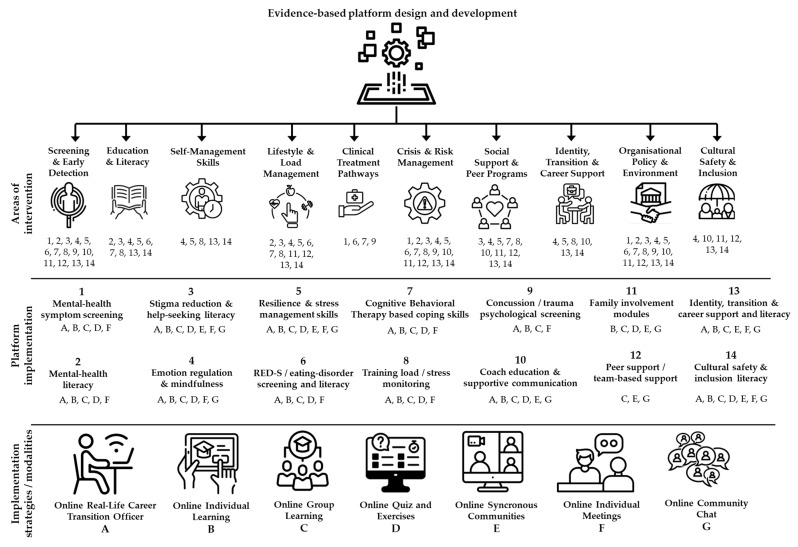
A harmonized framework for the development and implementation of an online platform to support elite athletes’ mental health and career transitions.

Finally, Figure 2 represents a proposed harmonized structure of intervention domains, intervention-based categories through a digital tool, and possible intervention modalities. In particular, the figure presents the final integrated framework derived from the sequential synthesis of Matrices 1, 2 and 3. It visually represents the relational pathways from main identified higher-order intervention domains (e.g., Matrix 2, areas of intervention, first section of the figure, n = 10 items, such as Screening and Detection, Self-Management Skills, Social Support and Peer Programs, etc.) to possible digital implementation categories (e.g., Matrix 3, potential platform implementation, second section of the figure, n = 14 items, such as Mental Health Symptom Screening, Training Load-Stress Monitoring, Identity, Transition and Career Support and Literacy, etc.). This figure presents a possible operationalization of the integrative umbrella review findings by distinguishing evidence-informed components (e.g., first and second sections of the figure) from forward-looking digital opportunities for intervention modalities (e.g., possible implementation strategies/modalities, third section of the figure, n = 7 items labeled from A to G) as:

(A) The availability of a tailored Real-Life Career Transition Officer, equipped with relevant skills and competencies to assist elite athletes;
(B) Online individual learning modules, self-directed in nature, freely and openly available as Reusable Learning resources;
(C) Online group learning modules, which could adopt a synchronous structure to stimulate interactions;
(D) Online quizzes and exercises, to stimulate learning and engagement;
(E) Online synchronous communities that organize online events and meetings on selected relevant topics;
(F) Online individual meetings, allowing for a personalized support approach;
(G) Online asynchronous community spaces (e.g., a dedicated chat and/or forum).

This framework consolidates the cross-review evidence into a proposed coherent structure that links empirical risk patterns to intervention priorities and feasible digital solutions. Therefore, it could serve as the operational foundation for the PORTAL project’s digital support model.

## 4. Discussion and Conclusions

The present integrative umbrella review provides an updated, comprehensive, and systematic overview of the most recent scientific evidence on mental health and career transition support challenges and needs among elite athletes, with a particular focus on sustainable, long-term solutions that could be delivered through innovative, cross-national digital solutions. The multidimensional literature analyzed in this study illustrates the multifaceted nature of elite athletes’ post-sport trajectories, the interplay of risk and protective factors, and the progressive shift toward holistic models of athlete development. Within this context, effective support cannot rely on single intervention domains. Despite the documented growing body of knowledge in this field [3], the present integrative umbrella review reveals a persistent absence of practical, scalable, and internationally coordinated tools capable of translating evidence into everyday support. This result is in line with recent studies in this field [10,26], reporting limited service provision across several national contexts, limited access to external professionals, and crisis-intervention and peer-support mechanisms remaining underdeveloped. This result also strengthens the concern that athlete mental health remains insufficiently integrated into organizational structures, often addressed reactively rather than proactively [16,20]. This gap emphasizes the urgent need for evidence-based, updated, multidimensional digital resources that can be adapted across cultural contexts and integrated into existing support systems to enhance the reach, continuity, accessibility, and sustainability of athletes’ support throughout and beyond their competitive careers. Based on a comprehensive evaluation of the existing literature in this field, the present integrative umbrella review also represents an original attempt to: (i) provide a comprehensive, evidence-based overview of the existing information on major intervention domains and possible strategies collected through the analysis of manuscripts on different research areas, in line with the multidimensional nature of effective, long-term elite athletes’ career assistance; (ii) highlight the relevance of each recorded intervention domain through a rigorous methodology, encompassing a systematic and reliable approach to data extraction and synthesis; and (iii) propose a harmonized and comprehensive model for the development and implementation of digital support solutions, comprehensive of major service domains and potential intervention modalities. Therefore, the present findings may be considered a useful tool in nurturing the sustainable growth of mental health and career transition assistance in the field of elite sport and in providing useful insights to the relevant stakeholders in the sector.

Regarding the quality appraisal process, an overall good quality of assessed manuscripts through specific tools applied in relation to study typology emerged, with issues arising for AMSTAR 2 scores only. Hence, the predominance of “critically low” AMSTAR-2 ratings requires additional interpretation. In fact, most of the SLRs reported issues in several appraisal items, suggesting a gap in methodological approaches and reporting rigor within this broad research area. Therefore, in this study, low AMSTAR 2 scores should be interpreted with caution, as AMSTAR-2 is optimized for clinical intervention reviews [36] and includes criteria that are structurally absent and/or not consistently applied in sport psychology and social science reviews [38]. Conversely, the substantial positive evaluations obtained through QATR, JBI, SANRA, and AGREE II indicate that the included manuscripts met methodological expectations within their respective paradigms. Accordingly, confidence in the synthesis was informed primarily by QATR outcomes for SLRs, and by both QATR and JBI score for ScRs. Additionally, moderate- to strong-quality conceptual and narrative sources contributed to theoretical and contextual insights, whereas good-quality consensus statements provided expert-derived recommendations.

Regarding the overlap analysis, although the overall CCA was low [49,50], several influential primary studies appeared repeatedly across multiple reviews. This concentration of evidence may have amplified certain themes (e.g., particularly those related to identity foreclosure, mental health symptomatology, and dual-career benefits), while under-representing less frequently studied domains. Such patterns are common in emerging fields, where a limited number of foundational studies may exert disproportionate influence. In addition to amplifying certain themes, the repeated citation of a small cluster of influential studies may also have shaped the architecture of the thematic map itself, as umbrella reviews inherently privilege patterns that appear consistently across sources. Hence, convergence around topics such as identity foreclosure or mental health symptomatology may partly reflect structural citation patterns rather than true prevalence or conceptual centrality in the broader evidence base. Accordingly, to avoid over-generalization, dominant themes were interpreted with caution, recognizing that convergence may partly reflect structural citation patterns rather than the true distribution of evidence across all primary studies.

The findings of this integrative umbrella review provide a higher-order synthesis showing that sustainable athlete career pathways and mental health support must be conceptualized as intertwined, lifespan processes shaped by identity dynamics, dual-career engagement, organizational environments, and access to culturally sensitive psychological services, thereby extending and consolidating the fragmented evidence identified in our previous systematic review of primary studies [3]. Consistent with foundational work on athletic retirement and career transitions [4,5], the included review, conceptual, and position studies converge on the view that retirement from elite sport is a complex, multifactorial transition involving profound changes in identity, mental health, social belonging, and vocational direction. This aligns with theoretical frameworks emphasizing the centrality of athletic identity and the risks of identity foreclosure [13,14], which our results further substantiate through review-level evidence documenting heightened psychological vulnerability when athletes define themselves primarily through their sporting role and face abrupt or involuntary retirement [55,67,71,74,76]. At the same time, diversified identities, dual-career engagement, and proactive career planning emerge as robust protective factors that facilitate smoother transitions and enhance long-term well-being [1,12,15,17,24,25,57,72,88,89,96,100,101,102,103], reinforcing calls for sustainable career pathways embedded within broader sustainability debates in sport and society [6,7,8]. Therefore, effective multi-level support mechanisms should be implemented, to offer elite athletes a wide range of services (e.g., mental health, well-being, lifestyle and self-management skills, career planning, coping skills, education and literacy relative to major thematic trends in this field, community-based and peer support) through sustainable digital interventions that could work in the long-term beyond national and organizational boundaries.

Across the included studies, mental health appears as both a determinant and an outcome of career sustainability. Echoing consensus statements in this field [16,20], the present integrative umbrella review confirms that elite athletes experience symptoms of anxiety, depression, disordered eating, burnout, and psychological distress at rates comparable to or exceeding those of the general population [16,17,55,61,71,80,82]. These vulnerabilities intensify during periods of uncertainty (e.g., injury, overtraining, deselection, performance decline, and especially retirement) where athletes often lose access to structured support and face difficulties reconstructing purpose and belonging [4,18,19,66,75,81,85]. Our synthesis also highlights the importance of contextual and structural determinants, including organizational culture, coaching practices, safeguarding failures, and gendered and disability-specific stressors [63,65,75,83,91], thereby reinforcing the argument that mental health in elite sport cannot be reduced to individual resilience or pathology but must be understood within a broader ecology of risk and protection. Confirming the previous literature in this field [10,26] documenting that mental health support services are among the most widely acknowledged but inconsistently implemented, with assessment practices remaining underdeveloped in many national contexts, possible implementation of digital resources and solutions should: (i) consider mental health major evidence-based trends; (ii) provide education and literacy to elite athletes and their surrounding support stakeholders; and (iii) implement online screening and early detection mechanisms through validated tools and direct interaction with professional, dedicated support providers (e.g., mental health, real-life, and career assistance experts).

Methodologically, the present integrative umbrella review reveals both progress and limitations in the review-level evidence base. Systematic and scoping reviews have proliferated since 2015, particularly in Europe and North America, reflecting growing recognition of mental health and transition issues in elite sport. However, quality appraisal indicates variability in methodological rigor, with some reviews lacking transparent search strategies, explicit eligibility criteria, or robust synthesis procedures. However, the integration of different appraisal methodologies and the applied rigorous evaluation and coding approach highlighted an overall good confidence of evidence and a minimal bias in data extraction and synthesis. Furthermore, overlap analysis shows that a relatively small cluster of primary studies recur across multiple reviews, increasing their influence on the evidence base. While this is not inherently problematic, it underscores the need for more diverse, longitudinal, and culturally inclusive primary research, particularly in under-represented populations such as paralympic athletes, athletes from the Global South, and those navigating non-traditional or precarious career pathways [3,10,26,66,75]. In particular, the evidence base included in this integrative umbrella review is characterized by notable geographical imbalance, with most studies originating from Europe, North America, and Australia. Research from Africa, Asia, South America, and the Middle East remains limited, constraining the global applicability of the findings. Representation gaps also emerged across athlete subgroups. Evidence on sex and gender differences is inconsistent and/or not specifically addressed within the included studies. Furthermore, para-athletes remain significantly under-represented despite their unique mental health needs. Finally, most studies focus on well-resourced elite sport systems, offering limited insight into socioeconomic inequalities or the experiences of athletes in lower-resource contexts. These limitations highlight the need for more inclusive, culturally diverse, and socioeconomically sensitive research to validate and refine the proposed framework.

Despite these limitations, the present integrative umbrella review identifies clear, convergent themes that advance the field beyond fragmented subdomains. Matrix 1 (Table 3) shows that mental health risk and protection are inseparable from the broader career ecology; symptoms, stressors, coping skills, identity processes, and contextual pressures interact across the lifespan, with retirement emerging as a particularly sensitive phase requiring structured, multi-level support [55,84,85,98]. Matrix 2 (Table 4) delineates ten interconnected intervention domains (e.g., screening, education, psychological skills, load management, clinical pathways, crisis and safeguarding, support systems, identity and transitions, policy and governance, and cultural inclusion), demonstrating that effective support cannot be reduced to isolated programs but must be embedded within coherent organizational and policy frameworks [1,10,21,59,77,92,99]. Then, Matrix 3 (Table 5) translates these domains into platform-relevant intervention categories, showing that digital systems can operationalize evidence-based strategies through integrated screening and monitoring tools [4,71,83], modular education and literacy targeted to elite athletes and their relevant stakeholders on major contextual thematic areas [60,61,69,70,91], self-management and coping skill training [58,73,74,76,89], strengthened support systems [53,59,77,88,93], and identity-career development resources [56,57,72,96]. Finally, the proposed integrated framework represented in Figure 2 clarifies how the three matrices function as sequential analytical layers, translating review-level evidence into a structured, digitally implementable support model.

The present findings resonate strongly with broader discussions on digital transformation in sport and health [30], providing a complementary [3,10] solid evidence base for initiatives such as the ERASMUS+ PORTAL project aiming to develop a digital platform and a network of Real-Life Transition Officers to support elite athletes’ transitioning to post-sport life. By aligning conceptual insights from career and identity models, the empirical evidence on mental health risk and protection, and the intervention domains identified in this integrative umbrella review, digital and hybrid systems can be designed to deliver continuous, personalized, and context-sensitive support across the entire athletic career. At the same time, the persistent gaps highlighted in the present study (e.g., stigma, uneven access to services, structural inequities in dual-career provision, and limited implementation of existing guidelines) underscore that digital tools should not be considered a universal solution. They must be embedded within ethically grounded, well-resourced, and policy-supported ecosystems that recognize mental health and sustainable careers as core responsibilities of sport organizations, educational institutions, and policy bodies. Notably, the proposed digital framework integrates components supported by review-level evidence (e.g., screening, monitoring, literacy), expert-derived recommendations (e.g., interdisciplinary care, culturally sensitive support), and theoretically grounded digital opportunities (e.g., reflective tools, adaptive psychoeducation). Given the limited empirical research on digital interventions in elite sport, these digital components should be understood as forward-looking innovations aligned with the PORTAL project rather than established evidence-based practices.

In conclusion, by integrating review-level evidence across mental health, dual careers, and career transitions, this integrative umbrella review moves the field beyond fragmented insights and toward a transdisciplinary, lifespan-oriented framework for sustainable athlete development. It shows that sustainable transitions require not only individual coping resources but also coherent organizational structures, culturally attuned services, and long-term career development pathways that extend well beyond the competitive years. The synthesis highlights consistent vulnerabilities during career transitions and retirement, and identifies clear intervention priorities, including early screening, continuous monitoring, education and literacy, self-management skills, stakeholder-inclusive support systems, and structured identity-career development. However, several limitations should be acknowledged.

First, the included reviews varied in quality, with inconsistencies in search strategies, conceptual definitions, and synthesis methods, and the overlap analysis revealed that a subset of primary studies disproportionately influenced the evidence base.

Second, appraisal results should inform the confidence with which findings are synthesized. In the present study, AMSTAR 2 ratings indicated that 78% of SLRs were of “critically low” or “low” quality, which might reduce the certainty of findings. This reflects, in part, the tool’s clinical orientation [36] not aligning with methodological norms in sport psychology and social science reviews [38]. In considering the positive scores derived from the QATR evaluation and the low application specificity of AMSTAR 2 in the field of the present study, AMSTAR 2 appraisal was not considered as a critical issue influencing the certainty of findings and derived conclusions. Conversely, QATR scores were considered more suitable to reflect the methodological rigor and quality of studies within this broader research area. Notably, to enhance the standards of quality and rigor of appraisal procedures in this research domain, the development of tailored appropriate tools could be envisioned in future research. In fact, whilst AMSTAR 2 confirmed a low application specificity in this context, the authors believe that: (i) the study designs and methodological rigor of experimental procedures and reporting standards should be enhanced; and (ii) the development of rigorous appraisal tools, suitable for the application in the field of sport, psychology and social sciences should be envisioned, to enhance both the rigor of the appraisal and methodological standards of studies. Aside appraisal specific considerations, the convergence across reviews suggests recurring themes in athlete mental health, identity, and career transitions. For these reasons, the proposed framework should be considered as an evidence-informed model that integrates empirical findings with expert-based recommendations and conceptual insights, rather than a definitive evidence-based guideline.

Third, the heterogeneous nature of the included studies might constitute a methodological limitation that moderates the certainty of drawn conclusions. It should also be acknowledged that the heterogeneity in athletic populations considered in the included manuscripts, sport contexts, and national systems further limits the generalizability of findings. In particular, the emerged publication bias, geographical imbalance, and sex/gender differences, para-athlete populations, and socioeconomic inequalities insufficiently represented limit the generalizability of the findings and underscore the need for more inclusive and globally diverse research in this area.

Fourth, the exclusion of student athlete populations limits the generalizability of findings related to educational engagement. While dual-career pathways are relevant across competitive levels, the present review focused exclusively on elite athletes to align with the scope of the PORTAL project and our previous SLR work [3].

Fifth, despite the slight CCA value, overlap may still reduce evidence independence, as several highly cited primary studies recur across multiple included studies. This may contribute to thematic dominance in areas such as mental health prevalence, identity foreclosure, and retirement adjustment. Although these patterns were considered when interpreting convergence across reviews, overlap should be acknowledged as a potential limitation in the present study.

Sixth, although the thematic synthesis followed a structured hybrid approach with multi-reviewer consensus, no statistical intercoder reliability coefficient was calculated. This reflects common practice in qualitative evidence synthesis but should be acknowledged as a methodological limitation.

Seventh, the narrative synthesis applied in the present study is primarily descriptive, reflecting the need for transparency across heterogeneous evidence types. Higher-order analytical integration is provided through the implemented structured matrices presented in this study, which consolidate cross-study patterns. Nonetheless, we acknowledge that a more extensive interpretive synthesis could further enrich the analysis.

Finally, although the proposed framework is grounded in review-level evidence, some digital implementation components remain theoretical due to limited empirical research on digital interventions in elite sport. In fact, empirical evidence on digital interventions for elite athletes is still emerging. As such, the digital components of the proposed framework represent theoretically grounded opportunities and are presented as evidence-informed opportunities rather than validated solutions and/or possible interventions. Future research is needed to evaluate the feasibility, acceptability, and effectiveness of digital support tools in high-performance sport contexts.

Despite these constraints, the present integrative umbrella review offers convergent insights with important practical implications. Sport organizations, educational institutions, and policy bodies should prioritize integrated, multi-level support structures that extend across the entire athletic career, ensuring equitable access to mental health services, dual-career opportunities, and transition planning. In this framework, digital resources and hybrid models may offer a promising avenue for operationalizing these recommendations by providing scalable, personalized, cross-national, and continuous support before, during, and after retirement. By aligning evidence, policy, and digital innovation, the sector can move toward more sustainable, ethically grounded systems that promote athlete well-being and long-term career development. In this context, by explicitly linking risk domains to intervention domains and digital implementation categories, the proposed framework provides a structured pathway for designing scalable, cross-national digital support systems aligned with the PORTAL project’s objectives. However, future research should evaluate the feasibility, acceptability, and effectiveness of digital and hybrid support systems across diverse athlete populations and cultural contexts.

## Figures and Tables

**Table 1:** An overview of the general characteristics of the included studies.

Variables	Frequency of Occurrence (n)			
Typology	Assessed	Excluded	Included	Exclusion Reasons
Position statements	8	1	7	Out of scope: psychiatric disorders (n = 1)
Conceptual	19	4	15	Out of scope: focused on religion and spirituality in career retirement (n = 1); focused on support towards sport performance (n = 1); focused on the career journey of a specific para-athlete population (n = 1); mostly focused on dual career (n = 1)
Systematic and scoping: SLR, ScR, MA, SR, UR *	30	6	24	Out of scope: mostly focused on athletic identity (n = 1); mostly focused on COVID-19 (n = 1). Not in English language (n = 1). Missed duplicate (n = 3)
Narrative reviews	10	4	6	Out of scope: focused mostly on sport performance (n = 1); depression on a specific athletic population (n = 1); psychiatric disorders (n = 1). Out of temporal range (n = 1)
Total	67	13	52	
**Geographical region**				
Australia			8	
Europe (most represented: Sweden n = 6; United Kingdom n = 8)			23	
South America (Brazil)			3	
North America (USA n = 6; Canada n = 8)			14	
Africa (South Africa)			1	
Asia (China n = 2; Israel)			3	
**Year of publication**				
2015			1	
2016			3	
2017			1	
2018			3	
2019			6	
2020			4	
2021			9	
2022			5	
2023			7	
2024			12	
2025			1	
**Research area**				
Career transitions, assistance and support			12	
Dual-career support			1	
Eating disorders			2	
Mental health support			34	
Well-being in elite athletes			3	

Note: * SLR = systematic literature review; ScR = scoping review; MA = meta-analysis; SR = systematic review; UR = umbrella review.

**Table 3:** Matrix 1, presenting the five identified higher-order themes.

Study	Code	Symptoms & Risk	Stressors	Transitions & Identity	Help-Seeking & Culture	Protective & Support
Schinke et al. 2015	[52]	1	1	1	1	1
Knights et al. 2016	[4]	1	0	1	0	1
Rice et al. 2016	[55]	1	1	1	1	1
Stambulova 2016	[57]	0	0	1	0	1
Schinke et al. 2017	[59]	1	1	1	1	1
Mannes et al. 2018	[61]	1	0	1	1	1
Moesch et al. 2018	[63]	1	1	0	0	1
Souter et al. 2018	[65]	1	1	1	1	1
Buckley et al. 2019	[67]	1	0	1	0	1
Castaldelli-Maia et al. 2019	[69]	1	0	0	1	1
Gouttebarge et al. 2019	[71]	1	1	1	0	0
Stillman et al. 2019	[73]	1	1	0	1	1
Swartz et al. 2019	[75]	1	1	1	1	1
Van Slingerland et al. 2019	[77]	1	1	0	1	1
Giles et al. 2020	[79]	0	1	0	0	1
Gorczynski et al. 2020	[81]	1	1	0	1	1
Henriksen et al. 2020	[21]	1	1	1	1	1
Küttel & Larsen 2020	[84]	1	1	0	0	1
Barth et al. 2021	[86]	1	0	1	0	1
Ekengren et al. 2021	[88]	0	1	1	0	1
Larsen et al. 2021	[90]	1	1	1	1	1
Perry et al. 2021	[91]	1	0	0	1	1
Poucher et al. 2021	[93]	1	1	0	1	1
Si et al. 2021	[94]	1	1	0	1	1
Stambulova et al. 2021	[96]	0	0	1	0	1
Vella et al. 2021	[98]	1	1	1	1	1
Wendling & Sagas 2021	[53]	1	0	1	0	1
Chen & Bansal 2022	[54]	1	0	1	0	1
Chroni & Dieffenbach 2022	[56]	1	1	1	1	1
Colagrai et al. 2022	[58]	1	0	0	0	1
Delfin et al. 2022	[60]	1	1	0	1	1
Purcell et al. 2022	[62]	1	1	0	1	1
Assa & Reizer 2023	[64]	1	0	1	1	1
Crossen et al. 2023	[66]	0	1	1	1	1
Nuetzel 2023	[68]	1	1	0	0	1
Pena-Pérez & Portela-Pino 2023	[70]	1	1	1	0	1
Rebelo et al. 2023	[72]	0	1	0	0	1
Voorheis et al. 2023	[74]	1	0	1	0	1
Waud & Weese 2023	[76]	1	0	1	1	1
de Oliveira Camilo et al. 2024	[78]	1	1	0	1	1
Fatt et al. 2024	[80]	1	1	1	0	1
FattMClinPsy et al. 2024	[82]	1	1	1	1	1
Gill et al. 2024	[83]	1	1	1	1	1
Gorczynski et al. 2024	[85]	1	1	0	1	1
Kusssman & Choo 2024	[87]	1	1	0	1	1
Prior et al. 2024	[89]	1	1	0	1	1
Runacres & Marshall 2024	[51]	1	1	1	0	1
Schinke et al. 2024	[92]	1	1	1	1	1
Stambulova et al. 2024	[1]	1	1	1	1	1
Stevens et al. 2024	[95]	1	1	0	0	1
Wylleman 2024	[97]	0	1	1	0	1
Li et al. 2025	[99]	1	1	0	1	1
**Total (n)**		**45**	**38**	**31**	**31**	**51**
Frequency of occurrence (%) *		**86.5**	**73.1**	**59.6**	**59.6**	**98.1**

Note: 1 = present in the manuscript; 0 = absent; * relative to the 52 included manuscripts.

**Table 4:** Matrix 2, presenting the ten identified intervention areas.

Study	Code	Category of Interventions									
		Screening & Early Detection	Education & Literacy	Self- Management Skills	Lifestyle & Load Management	Clinical Treatment Pathways	Crisis & Risk Management	Social Support & Peer Programs	Identity, Transition & Career Support	Organizational Policy & Environment	Cultural Safety & Inclusion
Schinke et al. 2015	[52]	1	1	1	1	1	1	1	1	1	1
Knights et al. 2016	[4]	0	1	1	0	0	0	1	1	0	0
Rice et al. 2016	[55]	1	1	1	1	1	1	1	1	1	1
Stambulova 2016	[57]	0	1	0	0	0	0	1	1	0	0
Schinke et al. 2017	[59]	1	1	1	1	1	1	1	1	1	1
Mannes et al. 2018	[61]	1	1	1	0	0	0	1	1	0	1
Moesch et al. 2018	[63]	1	1	1	1	0	0	1	0	1	0
Souter et al. 2018	[65]	1	1	1	1	1	1	1	1	1	1
Buckley et al. 2019	[67]	0	1	1	0	0	0	1	1	0	0
Castaldelli-Maia et al. 2019	[69]	1	1	0	0	0	1	1	0	1	1
Gouttebarge et al. 2019	[71]	1	1	0	1	1	1	0	0	1	0
Stillman et al. 2019	[73]	1	1	1	1	1	1	1	0	1	1
Swartz et al. 2019	[75]	1	1	1	1	1	1	1	1	1	1
Van Slingerland et al. 2019	[77]	1	1	1	1	1	1	1	0	1	1
Giles et al. 2020	[79]	0	1	0	1	0	0	1	0	1	1
Gorczynski et al. 2020	[81]	1	1	1	1	1	1	1	0	1	1
Henriksen et al. 2020	[21]	1	1	1	1	1	1	1	1	1	1
Küttel & Larsen 2020	[84]	0	1	0	1	0	0	1	0	1	0
Barth et al. 2021	[86]	0	1	1	0	0	0	1	1	0	0
Ekengren et al. 2021	[88]	0	1	0	1	0	0	1	1	0	1
Larsen et al. 2021	[90]	1	1	1	1	1	1	1	1	1	1
Perry et al. 2021	[91]	0	1	1	0	0	0	1	0	1	1
Poucher et al. 2021	[93]	1	1	1	1	1	1	1	0	1	1
Si et al. 2021	[94]	1	1	1	1	1	1	1	0	1	1
Stambulova et al. 2021	[96]	0	1	0	0	0	0	1	1	0	0
Vella et al. 2021	[98]	1	1	1	1	0	0	1	1	1	1
Wendling & Sagas 2021	[53]	0	1	1	0	0	0	1	1	0	0
Chen & Bansal 2022	[54]	0	1	1	0	0	0	1	1	0	1
Chroni & Dieffenbach 2022	[56]	1	1	1	1	1	1	1	1	1	1
Colagrai et al. 2022	[58]	0	1	0	0	0	0	1	0	0	1
Delfin et al. 2022	[60]	1	1	1	0	0	0	1	0	1	1
Purcell et al. 2022	[62]	1	1	1	1	1	1	1	0	1	1
Assa & Reizer 2023	[64]	0	1	1	0	0	0	1	1	0	1
Crossen et al. 2023	[66]	0	1	1	0	0	0	1	1	0	1
Nuetzel 2023	[68]	0	1	1	0	0	0	1	0	0	0
Pena-Pérez & Portela-Pino 2023	[70]	1	1	1	1	0	0	1	1	1	0
Rebelo et al. 2023	[72]	0	1	0	1	0	0	1	0	0	1
Voorheis et al. 2023	[74]	1	1	1	0	0	0	1	1	0	1
Waud & Weese 2023	[76]	1	1	1	0	0	0	1	1	0	1
de Oliveira Camilo et al. 2024	[78]	1	1	1	0	0	0	1	0	1	1
Fatt et al. 2024	[80]	1	1	1	0	1	1	1	1	1	1
FattMClinPsy et al. 2024	[82]	1	1	1	1	1	1	1	1	1	1
Gill et al. 2024	[83]	1	1	1	1	1	1	1	1	1	1
Gorczynski et al. 2024	[85]	1	1	1	1	1	1	1	0	1	1
Kusssman & Choo 2024	[87]	1	1	1	1	1	1	1	0	1	1
Prior et al. 2024	[89]	1	1	1	1	1	1	1	0	1	1
Runacres & Marshall 2024	[51]	1	1	1	1	0	0	1	1	1	1
Schinke et al. 2024	[92]	1	1	1	1	1	1	1	1	1	1
Stambulova et al. 2024	[1]	1	1	1	1	1	1	1	1	1	1
Stevens et al. 2024	[95]	1	1	1	1	0	0	1	0	1	0
Wylleman 2024	[97]	0	1	0	1	0	0	1	1	1	1
Li et al. 2025	[99]	1	1	1	1	1	1	1	0	1	1
**Total**		**35**	**52**	**42**	**33**	**24**	**25**	**51**	**30**	**36**	**40**
Frequency of occurrence (%) *		**67.3**	**100**	**80.8**	**63.5**	**46.2**	**48.1**	**98.1**	**57.7**	**69.2**	**76.9**

Note: 1 = present in the manuscript; 0 = absent; * relative to the 52 included manuscripts.

**Table 5:** Matrix 3, presenting the 14 identified intervention categories through an online platform.

Study	Code	Category of Platform Implementation													
		Mental Health Symptom Screening	RED-S/Eating Disorder Screening	Concussion/Trauma Psychological Screening	Training Load/Stress Monitoring	Mental Health Literacy	Stigma Reduction & Help-Seeking Literacy	Cultural Safety & Inclusion Literacy	Coping Skills	Emotion Regulation & Mindfulness	Resilience & Stress-Management Skills	Peer Support/Team-Based Support	Coach Education & Supportive Communication	Family Involvement Modules	Identity, Transition & Dual-Career Support
Schinke et al. 2015	[52]	1	1	1	1	1	1	1	1	1	1	1	1	1	1
Knights et al. 2016	[4]	1	0	0	0	1	1	0	1	1	1	1	0	0	1
Rice et al. 2016	[55]	1	1	1	1	1	1	1	1	1	1	1	1	1	1
Stambulova 2016	[57]	1	0	0	0	1	1	1	1	1	1	1	0	0	1
Schinke et al. 2017	[59]	1	1	1	1	1	1	1	1	1	1	1	1	1	1
Mannes et al. 2018	[61]	1	0	0	0	1	1	1	1	1	1	1	0	0	1
Moesch et al. 2018	[63]	1	0	0	1	1	1	1	1	1	1	1	0	0	1
Souter et al. 2018	[65]	1	1	1	1	1	1	1	1	1	1	1	1	1	1
Buckley et al. 2019	[67]	1	0	0	0	1	1	0	1	1	1	1	0	0	1
Castaldelli-Maia et al. 2019	[69]	1	1	0	0	1	1	1	1	1	1	1	0	0	1
Gouttebarge et al. 2019	[71]	1	0	1	1	1	1	0	1	1	1	0	0	0	0
Stillman et al. 2019	[73]	1	1	1	1	1	1	1	1	1	1	1	1	1	1
Swartz et al. 2019	[75]	1	1	1	1	1	1	1	1	1	1	1	1	1	1
Van Slingerland et al. 2019	[77]	1	1	1	1	1	1	1	1	1	1	1	1	1	1
Giles et al. 2020	[79]	1	0	0	1	1	1	1	1	1	1	1	0	0	1
Gorczynski et al. 2020	[81]	1	1	1	1	1	1	1	1	1	1	1	1	1	1
Henriksen et al. 2020	[21]	1	1	1	1	1	1	1	1	1	1	1	1	1	1
Küttel & Larsen 2020	[84]	1	0	0	1	1	1	1	1	1	1	1	0	0	1
Barth et al. 2021	[86]	1	0	0	0	1	1	1	1	1	1	1	0	0	1
Ekengren et al. 2021	[88]	1	0	0	1	1	1	1	1	1	1	1	1	0	1
Larsen et al. 2021	[90]	1	1	1	1	1	1	1	1	1	1	1	1	1	1
Perry et al. 2021	[91]	1	0	0	0	1	1	1	1	1	1	1	0	0	1
Poucher et al. 2021	[93]	1	1	1	1	1	1	1	1	1	1	1	1	1	1
Si et al. 2021	[94]	1	1	1	1	1	1	1	1	1	1	1	1	1	1
Stambulova et al. 2021	[96]	1	0	0	0	1	1	1	1	1	1	1	0	0	1
Vella et al. 2021	[98]	1	0	0	1	1	1	1	1	1	1	1	1	0	1
Wendling & Sagas 2021	[53]	1	0	0	0	1	1	1	1	1	1	1	0	0	1
Chen & Bansal 2022	[54]	1	0	0	0	1	1	1	1	1	1	1	0	0	1
Chroni & Dieffenbach 2022	[56]	1	1	1	1	1	1	1	1	1	1	1	1	1	1
Colagrai et al. 2022	[58]	1	0	0	0	1	1	1	1	1	1	1	0	0	0
Delfin et al. 2022	[60]	1	0	0	0	1	1	1	1	1	1	1	0	0	0
Purcell et al. 2022	[62]	1	1	1	1	1	1	1	1	1	1	1	1	1	1
Assa & Reizer 2023	[64]	1	0	0	0	1	1	1	1	1	1	1	0	0	1
Crossen et al. 2023	[66]	1	0	0	0	1	1	1	1	1	1	1	0	0	1
Nuetzel 2023	[68]	1	0	0	0	1	1	1	1	1	1	1	0	0	0
Pena-Pérez & Portela-Pino 2023	[70]	1	0	0	1	1	1	1	1	1	1	1	0	0	1
Rebelo et al. 2023	[72]	1	0	0	1	1	1	1	1	1	1	1	0	0	1
Voorheis et al. 2023	[74]	1	0	0	0	1	1	1	1	1	1	1	0	0	1
Waud & Weese 2023	[76]	1	0	0	0	1	1	1	1	1	1	1	0	0	1
de Oliveira Camilo et al. 2024	[78]	1	0	0	0	1	1	1	1	1	1	1	0	0	0
Fatt et al. 2024	[80]	1	1	1	1	1	1	1	1	1	1	1	1	1	1
FattMClinPsy et al. 2024	[82]	1	1	1	1	1	1	1	1	1	1	1	1	1	1
Gill et al. 2024	[83]	1	1	1	1	1	1	1	1	1	1	1	1	1	1
Gorczynski et al. 2024	[85]	1	1	1	1	1	1	1	1	1	1	1	1	1	1
Kusssman & Choo 2024	[87]	1	1	1	1	1	1	1	1	1	1	1	1	1	1
Prior et al. 2024	[89]	1	1	1	1	1	1	1	1	1	1	1	1	1	1
Runacres & Marshall 2024	[51]	1	0	0	1	1	1	1	1	1	1	1	1	0	1
Schinke et al. 2024	[92]	1	1	1	1	1	1	1	1	1	1	1	1	1	1
Stambulova et al. 2024	[1]	1	0	0	1	1	1	1	1	1	1	1	1	1	1
Stevens et al. 2024	[95]	1	0	0	1	1	1	1	1	1	1	1	0	0	1
Wylleman 2024	[97]	1	0	0	1	1	1	1	1	1	1	1	1	1	1
Li et al. 2025	[99]	1	1	1	1	1	1	1	1	1	1	1	1	1	1
**Total (n)**		**52**	**23**	**23**	**34**	**52**	**52**	**49**	**52**	**52**	**52**	**51**	**27**	**24**	**47**
Frequency of occurrence (%) *		**100**	**44.2**	**44.2**	**65.4**	**100**	**100**	**94.2**	**100**	**100**	**100**	**98.1**	**51.9**	**46.2**	**90.4**

Note: 1 = present in the manuscript; 0 = absent; * relative to the 52 included manuscripts.

**Table 6:** Hierarchical mapping across Matrix 1 (Needs), Matrix 2 (Intervention Domains), and Matrix 3 (Digital Implementation Components).

Matrix 1 Needs/Challenges	Matrix 2 Intervention Domains	Matrix 3 Potential Digital Components	Analytic Integration
**Symptoms & Risk**	Screening & Early Detection	Mental health symptom screening	Translate symptom-related needs into screening functions
		RED-S/eating disorder screening	Link between screening to sport-specific physiological and psychological risks
		Concussion/trauma psychological screening	Address trauma-related psychological risk within the elite athlete populations
	Lifestyle & Load Management	Training load/stress monitoring	Operationalize monitoring of stress-related risk factors
	Education & Literacy	Mental health literacy	Provide foundational knowledge to interpret symptoms and risks
**Stressors**	Lifestyle & Load Management	Training load/stress monitoring	Provide a direct response to performance- and environment-related stressors
	Education & Literacy	Stigma reduction & help-seeking literacy	Target cultural and cognitive barriers that exacerbate stress
	Cultural Safety & Inclusion	Cultural safety & inclusion literacy	Address contextual and environmental stressors linked to identity and belonging
	Self-Management Skills	Emotion regulation & mindfulness	Support coping with acute and chronic stressors
	Self-Management Skills	Resilience & stress-management skills	Build long-term adaptive capacity to manage stress
**Transitions & Identity**	Identity, Transition & Career Support	Identity, transition & dual-career support	Directly operationalize transition-related needs
	Self-Management Skills	Cognitive Behavioral Therapy based coping skills	Support identity reconstruction and adaptive coping
	Self-Management Skills	Emotion regulation & mindfulness	Facilitate emotional adjustment during transitions
	Self-Management Skills	Resilience & stress-management skills	Strengthen adaptive responses to uncertainty and change
**Help-Seeking & Culture**	Education & Literacy	Mental health literacy	Address literacy-related barriers to help-seeking
	Education & Literacy	Stigma reduction & help-seeking literacy	Directly target cultural and attitudinal barriers
	Social Support & Peer Programs	Peer support/team-based support	Provide relational pathways to help-seeking
	Organizational Policy & Environment	Coach education & supportive communication	Enhance supportive climates that facilitate help-seeking
**Protective & Support**	Social Support & Peer Programs	Peer support/team-based support	Reinforce protective interpersonal structures
	Organizational Policy & Environment	Coach education & supportive communication	Strengthen organizational protective factors
	Cultural Safety & Inclusion	Cultural safety & inclusion literacy	Enhance inclusion as a protective factor
	Clinical Treatment Pathways	Family involvement modules	Extend protective support to family systems
	Crisis & Risk Management	Concussion/trauma psychological screening	Ensure early detection of acute risks within protective frameworks

## Data Availability

No new data were created or analyzed in this study. Data sharing is not applicable to this article.

## Supplementary Materials

The following supporting information can be downloaded at https://www.mdpi.com/article/10.3390/sports14060251/s1, File S1: Prio checklist - protocol - search strings; File S2: Appraisal tools and overlap manuscripts; File S3: Full set of collected information for each assessed manuscript.
